# Towards a compact and precise sample holder for macromolecular crystallography

**DOI:** 10.1107/S2059798317013742

**Published:** 2017-09-29

**Authors:** Gergely Papp, Christopher Rossi, Robert Janocha, Clement Sorez, Marcos Lopez-Marrero, Anthony Astruc, Andrew McCarthy, Hassan Belrhali, Matthew W. Bowler, Florent Cipriani

**Affiliations:** a European Molecular Biology Laboratory, Grenoble Outstation, 71 Avenue des Martyrs, CS 90181, 38042 Grenoble, France

**Keywords:** sample holder, NewPin, miniSPINE, SPINEplus, high density, high precision, SmartMagnet

## Abstract

Most of the sample holders currently used in macromolecular crystallography offer limited storage density and poor initial crystal-positioning precision upon mounting on a goniometer. This has now become a limiting factor at high-throughput beamlines, where data collection can be performed in a matter of seconds. Furthermore, this lack of precision limits the potential benefits emerging from automated harvesting systems that could provide crystal-position information which would further enhance alignment at beamlines. This situation provided the motivation for the development of a compact and precise sample holder with corresponding pucks, handling tools and robotic transfer protocols. The development process included four main phases: design, prototype manufacture, testing with a robotic sample changer and validation under real conditions on a beamline. Two sample-holder designs are proposed: NewPin and miniSPINE. They share the same robot gripper and allow the storage of 36 sample holders in uni-puck footprint-style pucks, which represents 252 samples in a dry-shipping dewar commonly used in the field. The pucks are identified with human- and machine-readable codes, as well as with radio-frequency identification (RFID) tags. NewPin offers a crystal-repositioning precision of up to 10 µm but requires a specific goniometer socket. The storage density could reach 64 samples using a special puck designed for fully robotic handling. miniSPINE is less precise but uses a goniometer mount compatible with the current SPINE standard. miniSPINE is proposed for the first implementation of the new standard, since it is easier to integrate at beamlines. An upgraded version of the SPINE sample holder with a corresponding puck named SPINEplus is also proposed in order to offer a homogenous and interoperable system. The project involved several European synchrotrons and industrial companies in the fields of consumables and sample-changer robotics. Manual handling of miniSPINE was tested at different institutes using evaluation kits, and pilot beamlines are being equipped with compatible robotics for large-scale evaluation. A companion paper describes a new sample changer FlexED8 (Papp *et al.*, 2017, *Acta Cryst.*, D**73**, 841–851).

## Abstract 2

New compact and precise cryogenic sample holders for macromolecular crystallography are proposed as possible future European standards.

## 1. Introduction   

With the emergence of cryocrystallography (Teng, 1990 ▸) as a standard technique in macromolecular crystallography (MX), various sample holders for protein crystals were developed or adapted from existing supports for crystallographic cryogenic data collection (Garman & Owen, 2006 ▸). The ‘top-hat’ design, exemplified by the Hampton Research Magnetic Base, has proved to be highly successful and over many years has evolved into several similar designs that were subsequently standardized for the needs of robotic sample mounting (Cohen *et al.*, 2002 ▸; Karain *et al.*, 2002 ▸; Snell *et al.*, 2004 ▸; Cipriani *et al.*, 2006 ▸). Among them, the European SPINE standard was established in 2005 as an evolution of existing commercial cap-and-vial models. This standard played a key role in beamline automation in Europe and made it possible to collect data at different European beamlines with minimal compatibility problems (Beteva *et al.*, 2006 ▸). Nevertheless, as with all existing sample-holder standards, the SPINE standard has become a limiting factor at high-throughput beamlines.

At the most recent third-generation synchrotron MX beamlines, the time needed to centre and align a crystal with the X-ray beam is comparable to the time needed to collect an X-ray data set (Svensson *et al.*, 2015 ▸; Casanas *et al.*, 2016 ▸). This significantly impairs beamline efficiency. The situation will become worse at future fourth-generation light sources where only tens of milliseconds will be necessary for a typical MX data collection. This overhead could be significantly reduced by using sample holders that provide precise initial crystal positioning, in particular for crystals harvested by automated systems that can record a crystal position in the sample holder (Cipriani *et al.*, 2012 ▸; Deller & Rupp, 2014 ▸; Zander *et al.*, 2016 ▸). Improved initial crystal positioning will reduce crystal-alignment time for both optical (Lavault *et al.*, 2006 ▸; Pothineni *et al.*, 2006 ▸) and X-ray-based methods (Svensson *et al.*, 2015 ▸; Song *et al.*, 2007 ▸; Bowler *et al.*, 2016 ▸). Similarly, for serial data collection from microcrystals, the region of interest can be directly scanned after the sample holder has been mounted on the goniometer and the recorded alignment offset has been applied (Zander *et al.*, 2015 ▸; Gati *et al.*, 2014 ▸). An additional limiting factor is the size of the sample holders, as this directly impacts sample-storage density and the associated storage and transport costs. In Europe, most sample changers are based on six-axis industrial robotics and use SPINE or uni-puck (http://smb.slac.stanford.edu/robosync/Universal_Puck/) containers. Maximum storage density is currently obtained with the uni-puck (16 samples), allowing up to 112 samples (in seven uni-pucks) to be sent in a single CX100 shipping dewar. Modern high-throughput beamlines now use sample changers equipped with dewars that can hold up to 384 samples (24 uni-pucks; Bowler *et al.*, 2015 ▸; Nurizzo *et al.*, 2016 ▸; Owen *et al.*, 2016 ▸; Russi *et al.*, 2016 ▸). This capacity is obtained at the cost of using large sample-changer dewars. Storage density can also be increased by using specific containers such as the SSRL cassette used by the SAM sample changer (Cohen *et al.*, 2002 ▸; Russi *et al.*, 2016 ▸) that allow up to 192 Hampton Research CrystalCap-like sample holders to be placed in a CX100 shipping dewar. Nevertheless, SAM pucks have not been considered in Europe, probably because access from the side of the container is too different from the widespread top access and would require the adaptation of current robotic sample changers. Similarly, a proprietary high-density sample-holder system, which is used at the SPring-8 synchrotron, has been developed (Ueno *et al.*, 2004 ▸). The SPACE system is based on high-density plastic pins with two screw threads for transport and mounting on the goniometer. However, extending its usage to Europe would require the use of specific shipping dewars and a major integration effort at beamlines.

In common with other existing sample-holder standards, the European SPINE standard has two fundamental limitations. Firstly, the size of the sample holder limits the sample-storage density to 112 samples per transport dewar. Secondly, the mechanical tolerances and absence of an orientation index induce a variation of up to 1 mm in the initial crystal positioning at the sample position upon mounting. This also limits the repositioning precision upon loading/unloading, which makes loop and/or crystal alignment necessary before each data collection (Lavault *et al.*, 2006 ▸; Pothineni *et al.*, 2006 ▸; Svensson *et al.*, 2015 ▸). Furthermore, the cost of the sample holder can limit the number of samples prepared and therefore the usage of high-throughput techniques for synchrotron data collection (Abola *et al.*, 2000 ▸). Reusable sample-holder bases can contribute to cost reduction but require additional manpower to clean and recycle the sample holders. Consequently, in 2009 a feasibility study for a compact, precise and, as far as possible, cost-effective sample holder with corresponding manual and robotic handling tools compatible with six-axis industrial robots was initiated at the EMBL Grenoble Outstation. This project has been supported since 2011 by the BioStruct-X FP7 European programme, with the aim of defining a new European sample-holder standard. The kick-off meeting was held in December 2011 in Hamburg with the participation of seven partners: (i) SLS (Paul Scherrer Institute, Villigen, Switzerland); (ii) BESSY II (Helmholtz-Zentrum Berlin für Materialien und Energie, Berlin, Germany); (iii) MAX-IV laboratory (Lund, Sweden); (iv) EMBL@PETRA-III (European Molecular Biology Laboratory, Hamburg, Germany); (v) ESRF (The European Synchrotron Radiation Facility, Grenoble, France); (vi) DLS (Diamond Light Source Limited, Oxford, England) and (vii) EMBL Grenoble (European Molecular Biology Laboratory, Grenoble, France). The discussions conducted after the initial development stages revealed the importance of beamline-integration aspects and led to the definition of two new sample-holder designs, NewPin and miniSPINE, together with an improved version of the SPINE standard called SPINEplus. All of the corresponding containers are compatible with a specific sample-changer dewar slot that can also receive standard uni-pucks. Migration from the SPINE standard to NewPin, the ultimate sample-holder version proposed, could then be facilitated. All of the sample-holder models have been tested on a beamline under real conditions, using a sample changer built around a storage dewar (Papp *et al.*, 2017 ▸), a six-axis Stäubli TX60L industrial robot and corresponding grippers. The miniSPINE model was selected for large-scale testing as it is easier to integrate with existing beamline robotics and can be handled manually. 12 evaluation kits were manufactured and distributed to the project partners, other interested synchrotrons and institutes (IBS, NSLSII, Photon Factory and SPring-8) and to industrial companies working in the field. The feedback obtained concerning the ergonomics of the manual tools has been integrated into the final design of the mini­SPINE version. Here, we describe the design, testing and results from the use of the three sample holders proposed.

## 2. Experimental details   

**Figure 1:**
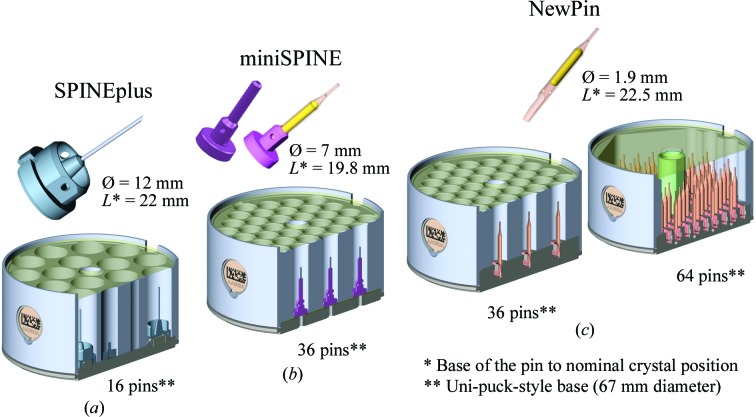
Overview of the presented sample holders with main dimensions and their corresponding 36-well pucks: (*a*) SPINEplus, (*b*) miniSPINE and (*c*) NewPin shown with a supplementary 64-well puck version.

The first phase of the development process focused on the NewPin sample holder, which is a simple pin of 22 mm in length and 1.9 mm in diameter. Its design fully meets the requirements for high storage density (36 samples per puck and up to 64 for the model anticipated for fully automated robotic pin handling) with a repositioning precision of 10 µm (Papp *et al.*, 2017 ▸). Nevertheless, after the initial evaluation it became clear that an intermediate version that was easier to handle manually and to integrate at beamlines would be required. Therefore, the miniSPINE sample holder was developed: a compact version of the SPINE sample holder that provides high storage density (36 samples per puck) and is easier to integrate on existing beamlines. The type of crystal mount (*i.e.* the loop or support that will hold the crystal) of the sample holders is not defined as they can receive any commercially available nylon loop/LithoLoop (Molecular Dimensions, Suffolk, England) or MicroMounts (MiTeGen, Ithaca, USA) or can be customized for specific requirements, such as for the CrystalDirect harvester (Cipriani *et al.*, 2012 ▸; Zander *et al.*, 2016 ▸). Attempts were made to design a vial for both new sample-holder types. Different vial-to-pin coupling methods were explored but insoluble handling problems, as well as the difficulties anticipated in manufacturing the vials at an affordable cost, led this option to be abandoned. Consequently, a closed robot gripper that acts as a cold buffer was developed to keep the crystals below 100 K during transfers in ambient air and to protect them from ice contamination. This gripper is compatible with both the NewPin and miniSPINE sample holders. Corresponding storage pucks and manual handling tools were also developed for the NewPin and miniSPINE sample holders. Both pucks can store 36 sample holders, leading to an increase in sample density in the widely used CX100 dry-shipping dewars by a factor of five *versus* the SC3-SPINE pucks (Cipriani *et al.*, 2006 ▸) and of more than two *versus* uni-pucks. The uni-puck footprint standard (http://smb.slac.stanford.edu/robosync/Universal_Puck) was adopted to ensure backwards compatibility with uni-pucks and to facilitate the migration of sample changers already installed at European MX beamlines, such as CATS (Jacquamet *et al.* 2009 ▸), G-Rob (Ferrer *et al.*, 2013 ▸), BART (Diamond Light Source, England) and ACTOR Rigaku systems (http://smb.slac.stanford.edu/robosync/). A specific dewar slot that is compatible with uni-pucks and that ensures precise positioning of the NewPin and miniSPINE pucks has also been developed. Furthermore, to offer an interoperable line of sample holders and pucks, we decided to slightly modify the SPINE standard, creating the SPINEplus sample holder and a corresponding sample-storage puck. The three new supports and associated pucks are shown in Fig. 1 ▸. The three sample-holder models are designed to be pre-oriented in specific storage racks to be further manipulated with known orientation (see §[Sec sec2.4]2.4).

Finally, it is possible to have an automated beamline environment that is compatible with both miniSPINE and SPINE sample holders, thus enabling a smooth transition between the actual SPINE standard, miniSPINE and subsequently NewPin. The following sections describe the new sample holders, their storage pucks and handling tools.

### 2.1. SPINEplus   

SPINEplus (Supplementary Fig. S1) has been designed to facilitate migration from the current SPINE standard to miniSPINE and from there to NewPin. The SPINEplus sample holder is backwards-compatible with the popular SPINE sample-holder standard. Its corresponding puck has a uni-puck footprint and is machine-identifiable. This is a key feature when different sample-holder models are used on the same beamline. The technical specifications are available in the Supporting Information.

#### 2.1.1. The SPINEplus sample holder   

**Figure 2:**
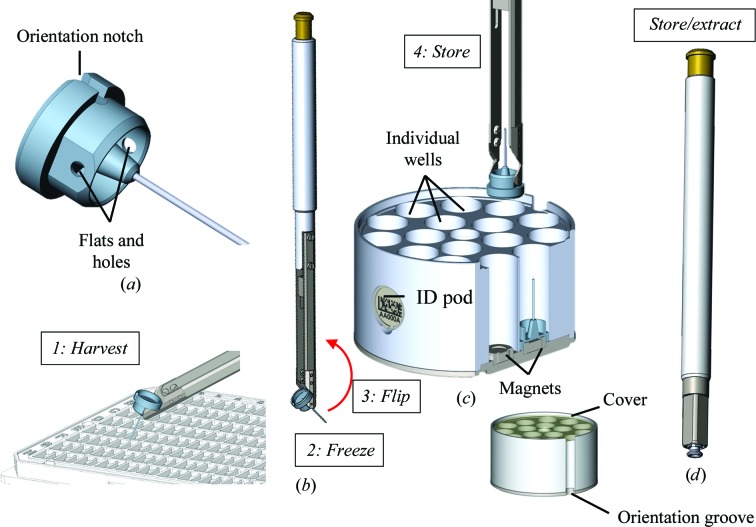
SPINEplus. (*a*) Sample holder with orientation notch, flats and holes for compatibility with the manual harvesting tool. (*b*) Harvesting tool. (*c*) Puck with identification pod, 16 individual pin wells, puck- and pin-holding magnets, cover and orientation groove. (*d*) Pin-extracting tool. Manual harvesting steps are indicated in italics.

Compared with the SPINE sample holder, SPINEplus (Fig. 2 ▸ *a*) includes the following new features: (i) an orientation slot at the base of the cap for orientation indexing on goniometers, in pucks or on automated harvesting systems; (ii) a tighter tolerance of the inner diameter of the cap for compatibility with new robotic tools and (iii) flats and holes on each side of the cap to grab, flip and store the sample holder in a SPINEplus puck after crystal harvesting using a manual harvesting tool (Fig. 2 ▸ *b*). Despite these modifications, the SPINEplus cap remains compatible with the SPINE vials and pucks, as well as with uni-pucks.

#### 2.1.2. The SPINEplus puck   

The SPINEplus puck (Fig. 2 ▸ *c*) has a capacity of 16 samples, permitting the storage of up to 112 sample holders in one CX100 dry-shipping dewar. It is composed of (i) a ferromagnetic stainless-steel base containing 16 magnets on the upper side to hold the pins, and four magnets on the bottom side to maintain the puck in a ferromagnetic dewar slot; (ii) an aluminium body with 16 wells to keep the sample in liquid nitrogen during transport, even when inclined; (iii) an identification pod to facilitate puck tracking and (iv) a nonferro­magnetic stainless-steel cover to protect crystals from ice contamination during transport. The pins are stored vertically with crystals at the top, locked by the magnets. The pins can be inserted into the puck wells (regardless of their angular orientation) using a specific manual harvesting tool (Fig. 2 ▸ *b*) or the gripper of a robotized system, such as a sample changer or the cryo­storage robotics of an automated crystal harvester (Papp *et al.*, 2017 ▸). The identification pod described in §[Sec sec2.5]2.5 combines human-readable and Datamatrix codes, and can receive an RFID tag. The SPINEplus puck fits into specific dewar slots that can also receive uni-puck, miniSPINE and NewPin pucks (Papp *et al.*, 2017 ▸).

#### 2.1.3. The SPINEplus handling tools   

Two manual tools have been developed to manipulate the SPINEplus sample holders: the crystal-harvesting tool (Fig. 2 ▸ *b*) to hold the pins during crystal harvesting and to store them in pucks, and the pin-extracting tool (Fig. 2 ▸ *d*) to remove the pins from the pucks. Both tools are used in a similar manner to the handling schemes adopted for NewPin and miniSPINE described in §[Sec sec2.2.4]2.2.4, except that no pin-grabbing or puck-loading assistants are necessary.

### 2.2. miniSPINE   

The miniSPINE design (Supplementary Fig. S3) enables the storage of up to 36 samples in a puck and can offer initial crystal positioning and positioning repeatability within 100 µm, depending on the transfer robotics used. The mini­SPINE sample holder is a compact, SPINE-type sample holder with a 7 mm diameter ferromagnetic base (Figs. 1 ▸ *b* and 3 ▸ *a*). Similar to the SPINE model, it is held on a goniometer with a magnetic mount. Both SPINE and miniSPINE can be used consecutively on a beamline using a special goniometer mount (Fig. 4*a* ▸ and Supplementary Fig. S6). miniSPINE has been designed for high storage density and has the advantage of easier integration compared with NewPin at beamlines equipped with goniometers that use magnets to hold the sample, in particular on those compatible with the SPINE sample-holder standard. Unlike NewPin, the positioning precision of the miniSPINE sample holder on a goniometer is highly dependent on the precision of the transfer robotics (Papp *et al.*, 2017 ▸). The technical specifications of the pins and puck are available in the Supporting Information.

#### 2.2.1. The miniSPINE sample holder   

**Figure 3:**
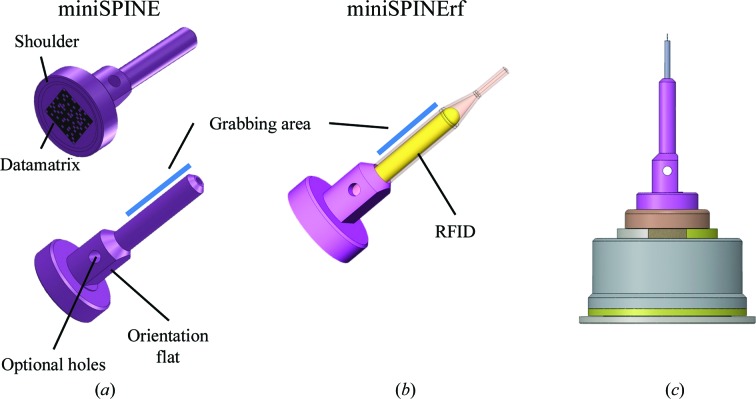
miniSPINE sample holder and goniometer mount. (*a*) Three-dimensional view of miniSPINE showing the optional Datamatrix, pin-grabbing area, orientation flat and optional holes for the manual harvesting tool. (*b*) Three-dimensional view of miniSPINErf shown with an RFID tag. (*c*) SmartMagnetP goniometer mount shown with a miniSPINE sample holder.

**Figure 4:**
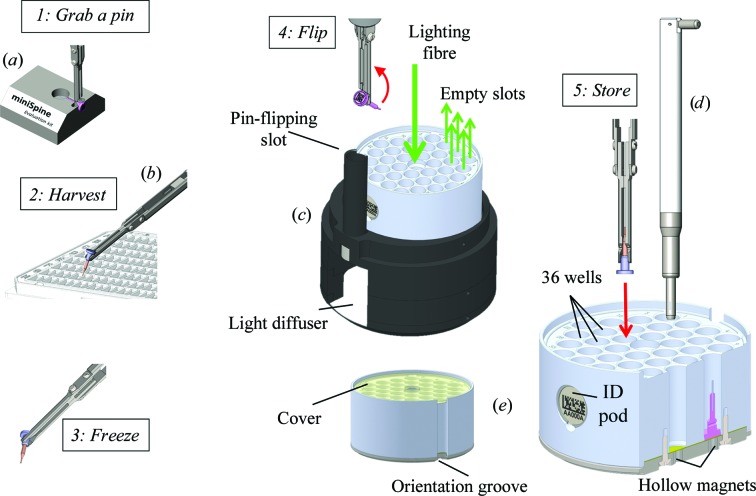
miniSPINE puck and manual handling tools. (*a*) Pin-grabbing assistant. (*b*) Manual harvesting tool. (*c*) Puck-loading assistant. (*d*) Pin-extracting tool (also compatible with the NewPin sample holder). (*e*) Puck with identification pod, 36 individual pin wells, puck- and pin-holding hollow magnets, cover and orientation groove. Manual harvesting steps are indicated in italics.

Two designs for the miniSPINE sample holder, miniSPINE (MS) and mini­SPINErf (MSrf) (Figs. 3 ▸ *a* and 3 ▸ *b*), are proposed. Mechanically compatible, they offer identification using either a Datamatrix label or an RFID tag. MS is a single piece of ferromagnetic metal that can receive different commercially available crystal mounts set on standard 0.63 mm needles, such as nylon loops, MicroMounts or LithoLoops. It can optionally be identified with a 14-point ECC200 Datamatrix code engraved on its base for tracking purposes. MSrf is composed of two parts: a base that is similar to the base of MS (shown in purple in Fig. 3 ▸ *a*), and a tube (Fig. 3 ▸ *b*, salmon) that is similar to the NewPin sample holder (§2.3). The MSrf model can host an RFID tag and directly receive crystal mounts on its 0.63 mm tip. The distance from the base of the miniSPINE sample holder to the crystal is fixed at 19.8 mm. This ensures compatibility with goniometer mounts designed to receive both SPINE and miniSPINE sample holders (§2.6). The optional holes on each side of the pin-support base (Figs. 3 ▸ *a* and 3 ▸ *b*) allow handling with a manual harvesting tool (Fig. 4*b* ▸) and the ability to flip between harvesting and storing positions (Fig. 4 ▸). Furthermore, when stored in a supply rack, the angular orientation of the miniSPINE sample holders can be fixed using the orientation flat (Fig. 3 ▸ *a*). This feature is essential when automated harvesting methods that require a fixed pin orientation are used (Cipriani *et al.*, 2012 ▸). A 0.2 mm shoulder at the base of the sample holder (Fig. 3 ▸ *a*) reduces the surface area of contact with the support (the bottom of the well in the puck or the goniometer) to a ring. This minimizes the effect of ice or particle contamination, thus improving the positioning precision and stability of the pins on goniometers and in pucks. The integrity of crystals during shipping and robot handling is ensured by individual wells in the pucks (Fig. 4 ▸ *e*) and by the closed robot grippers that act as cryo-tongs during sample transfer (Papp *et al.*, 2017 ▸). The robot grips miniSPINE supports by the 1.9 mm diameter section; therefore, the same robot gripper can be used to handle both the NewPin and miniSPINE sample holders (Papp *et al.*, 2017 ▸).

#### 2.2.2. The miniSPINE goniometer mount   

The miniSPINE sample holder, like the SPINE sample holder, is held magnetically on the goniometer. Although a simple permanent magnet is sufficient to hold a pin on a goniometer, to ensure reliable sample transfer, beamlines are usually equipped with electromagnets that both hold and detect sample holders, so-called SmartMagnets (Cipriani *et al.*, 2006 ▸). These devices were developed for the SPINE sample holder and have a concentric magnetic pole topology that is not compatible with miniSPINE. In this original SmartMagnet, the magnetic poles are coaxial. The first pole is in the centre and the second is a ring situated where the SPINE sample holders sit. When mounted, a SPINE cap closes the magnetic circuit, giving a sufficient holding force. However, this topology does not provide sufficient force to hold miniSPINE as it is only in contact with the central pole. Therefore, we have developed a new type of SmartMagnet with parallel magnetic poles, the SmartMagnetP (Supplementary Fig. S6), that is compatible with both SPINE and miniSPINE. As shown in Supplementary Fig. S6, the topology of the SmartMagnetP is such that the two poles of the electromagnet are parallel. In this configuration the magnetic circuit is closed for both SPINE and miniSPINE, providing sufficient holding force for both sample holders. The SmartMagnetP is geometrically and electrically interchangeable with the classical SmartMagnet, making a goniometer compatible with both SPINE and miniSPINE sample holders. The SPINE sample holder is guided and approximately centred by the central part of the SmartMagnet when mounted on the goniometer mount (§2.6), whereas the miniSPINE sample holder sits on the flat end of the SmartMagnetP (Fig. 3*c* ▸). Therefore, no mechanical centring is applied and the positioning precision of the miniSPINE pin primarily depends on the precision of the handling robotics. When manual mounting on a goniometer is necessary, for example when harvesting crystals at a beamline, an adaptor ring (not shown) can be mounted on the SmartMagnetP to facilitate mounting of the sample holder at the centre of the goniometer mount. Specific goniometer mounts with positioning stops could also be developed to improve the initial and repositioning precision of miniSPINE when compatibility with SPINE or SPINEplus sample holders is no longer necessary (Papp *et al.*, 2017 ▸).

#### 2.2.3. The miniSPINE sample-storage puck   

The miniSPINE puck (Fig. 4 ▸ *e*) can hold up to 36 miniSPINE sample holders in a format compatible with dry-shipping dewars. It is composed of (i) a ferromagnetic stainless-steel base with 36 hollow magnets to maintain the pins in position and to hold the puck in place when it is installed on a ferromagnetic dewar slot; (ii) an aluminium body with 36 wells that maintain the samples in liquid nitrogen during transfer of the puck between dewars; (iii) an identification pod to facilitate puck tracking and (iv) a nonferromagnetic stainless-steel cover to minimize ice contamination during transport in air. The pins are stored vertically with the crystals pointing up. A taper in the diameter at the bottom of each well enables precise positioning of the miniSPINE pins inside the puck upon insertion. The identification pod combines human-readable and Datamatrix codes, and can receive an RFID tag (Fig. 4 ▸ *e*). The miniSPINE puck is compatible with specific dewar slots that can also receive uni-puck, SPINEplus and NewPin pucks (Papp *et al.*, 2017 ▸). Up to 288 pins can be transported in a CX100 dewar using the eight-puck top-access canister or up to 252 using the seven-puck shelved canister (§2.7).

#### 2.2.4. The miniSPINE handling tools   

A set of manual tools have been developed to handle miniSPINE pins (Fig. 4 ▸ and Supplementary Fig. S5). In April 2015 miniSPINE evaluation kits were distributed to partner sites and industrial partners in order to assess the ergonomics of manual handling. Requests and suggestions from project partners were addressed and integrated to form the design presented here.

As miniSPINE is considerably smaller than the current mounts, a puck-loading assistant was developed (Fig. 4 ▸ *c*) to facilitate loading the pins into the puck during manual crystal harvesting (Supplementary Video S1). It is composed of a support with a reflective base installed in a dewar (26 B/BE, KGW Isotherm, Karlsruhe, Germany) and a light source equipped with an optical fibreglass light guide with a 7.8 mm diameter tip. The puck is placed in the support and the tip of the fibre is inserted in the centre of the puck. The dewar can be filled with liquid nitrogen before or after the loading assistant and miniSPINE puck are installed. The light emitted by the fibre is diffused by the bottom of the assistant and passes through the hollow magnets of the wells, clearly identifying wells that are free or occupied. Crystal harvesting (Fig. 4 ▸) starts by inserting an empty pin in the grabbing assistant (Step 1). Held by a magnet and properly oriented, the pin is placed in the harvesting tool and further rotated until it locks at 45° in a groove, the position used to harvest crystals from crystallization trays (Step 2). After harvesting and cryoprotection, the pin can be plunged into liquid nitrogen (Step 3) and then flipped using the pin-flipping slot of the puck-loading assistant located on the side of the puck slot (Step 4) below the liquid-nitrogen level, allowing the pin orientation to be changed to be placed into the puck. When aligned with the harvesting tool, the pin with crystal can be inserted into an empty puck slot (Step 5). It is important to vitrify the crystal before flipping. This ensures faster cooling and secures the attachment of the crystal in the cryo-mount. The level of liquid nitrogen in the dewar should be kept at a minimum of 5 cm above the flipping slot to ensure safe crystal handling. After processing, the pins can be removed from the puck using the pin-extracting tool (one pin at a time). The holes on the bottom of the pucks can also be used to simultaneously push all of the pins out of the pucks. The design of a pin-extracting tool with 36 fingers is shown in Supplementary Fig. S12. It can be used without the handle (fingers pointing up) with the puck installed above to safely recover each individual pin with tweezers, or with the handle to clear the puck.

### 2.3. NewPin   

**Figure 5:**
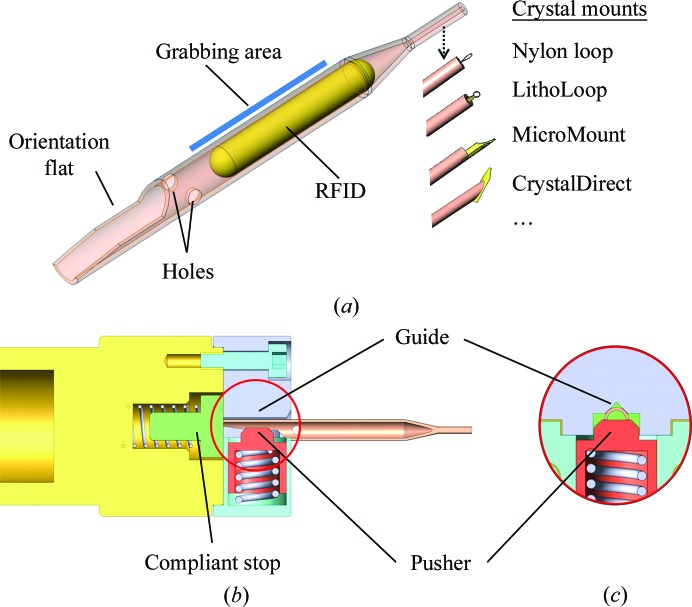
NewPin sample holder and goniometer mount. (*a*) Three-dimensional view of the NewPin sample holder with the orientation flat, two optional grabbing holes for the manual harvesting tool, the pin-grabbing area, an optional RFID tag and the 0.635 mm tip that can receive different crystal mounts. (*b*, *c*) Principle drawing of the goniometer mount that shows the compliant stop, the V-shaped guide and the orientation/locking pusher

**Figure 6:**
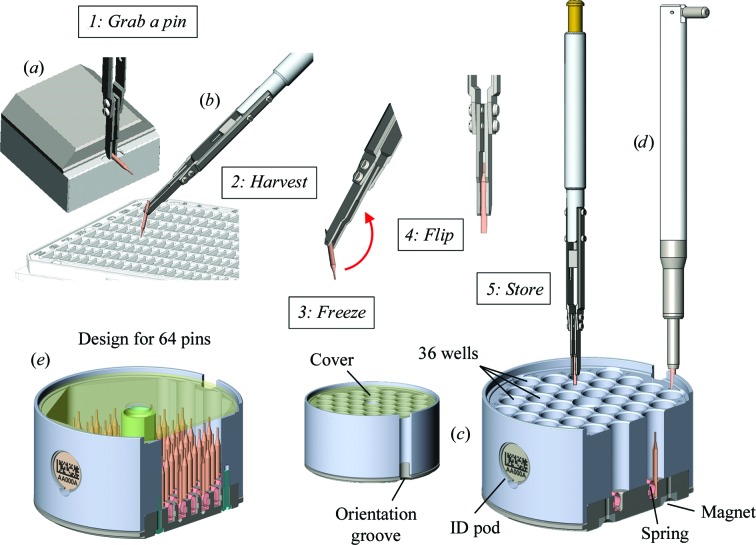
NewPin puck and manual handling tools. (*a*) Pin-grabbing support. (*b*) Harvesting tool. (*c*) Puck with identification pod, 36 individual pin wells, puck-holding magnets, pin-orienting/holding springs, cover and orientation groove. (*d*) Extracting tool (also compatible with miniSPINE sample holders). Manual harvesting steps are indicated in italics.

NewPin (Fig. 5 ▸ *a* and Supplementary Fig. S7) is our ultimate sample-holder proposition. The sample holder is a single pin on which the crystal mount is directly attached. Specifically adapted for fully automated harvesting and data-collection pipelines, it should fit perfectly into future entirely robotic systems that cover crystal harvesting and data collection. In this case, the storage density could reach up to 64 sample holders in a puck and up to 512 samples in the eight-puck top-access canister compatible with the CX100 shipping dewar. Manual crystal harvesting is difficult but possible using a set of handling tools and pucks with a capacity reduced to 36 sample holders currently proposed here. A possible design for a 64-sample version is proposed in Fig. 6 ▸(*e*). The NewPin sample holder fits in a specific gonio­meter mount containing a socket (Figs. 5 ▸ *b* and 5 ▸ *c*; Supplementary Fig. S8) that allows a crystal positioning repeatability of better than 10 µm. The technical specifications are available in the Supporting Information.

#### 2.3.1. The NewPin sample holder   

NewPin (Fig. 5 ▸ *a*) consists of a single needle of 22 mm in length and 1.9 mm in diameter, reducing to 0.64 mm diameter at one end to receive a cryo-loop or equivalent crystal support. At the other end of the needle, or pin base, a 4 mm flat plane is provided to fix the angular orientation on a goniometer mount, inside a puck and, potentially, on a crystal harvester mount. The bottom of the pin base is flat to fix its axial position. A specific auto-aligning and auto-orienting mechanical goniometer mount (§[Sec sec2.3.2]2.3.2) has been developed to reach a repositioning precision within 10 µm (Papp *et al.*, 2017 ▸). Two holes on the sides are provided to grab and flip the pin with a specific tool used for manual harvesting (§[Sec sec2.3.4]2.3.4). Optionally, the pin can be hollow to receive an RFID tag and facilitate sample tracking (§[Sec sec2.5]2.5). The grabbing area for the robot is situated on the 1.9 mm diameter part of the pin (Fig. 5 ▸ *a*). For the reasons previously explained, there is no vial associated with the NewPin sample holder. The length of the pin was chosen to facilitate integration and compatibility with existing gonio­meter mounts, and kappa goniometers, compatible with SPINE sample holders (§[Sec sec2.6]2.6). NewPin can be fabricated by machining, tube swaging or stamping. The last method is the most cost-effective for large production batches but requires a significant investment in machine tooling.

#### 2.3.2. The NewPin goniometer mount   

The NewPin gonio­meter mount (Figs. 5 ▸ *b* and 5 ▸ *c*) is a mechanical socket that automatically fixes the three-dimensional position of the pin upon insertion, with automatic correction of the initial positioning and orientation. The auto-aligning system tolerates an initial radial orientation error of up to ±5°. The socket consists of a V-shaped guide, an orienting/locking pusher and a compliant stop. In the prototype (Supplementary Fig S8), the compliant stop and orientation finger are each composed of a spring and a jack. For optimal three-dimensional positioning, the pins should be inserted pre-oriented within ±5° and about 0.5 mm further in than the nominal position. This ensures that upon release the compliant stop brings the pin back to the nominal axial position, while the orientating/locking pusher pushes it towards the V-shaped guide to ensure the correct radial and angular position. A crystal-repositioning precision better than 10 µm was obtained upon successive loading/unloading of the same pin (Papp *et al.*, 2017 ▸).

#### 2.3.3. The NewPin puck   

The NewPin puck (Fig. 6 ▸ *c*) can hold up to 36 NewPin sample holders in a format compatible with dry-shipping dewars. It is composed of (i) a ferromagnetic stainless-steel base containing 36 mechanical sockets to maintain the pins in position and four magnets to hold the puck in a ferromagnetic uni-puck dewar slot; (ii) an aluminium body with 36 wells to maintain the samples in liquid nitrogen during transport; (iii) an identification pod to facilitate puck tracking and (iv) a nonferromagnetic stainless-steel cover to minimize ice contamination during transport. The pins are stored vertically with crystals pointing up and locked in the sockets by locking/orienting springs (Fig. 6 ▸ *c*). The pins are plugged pre-oriented using a specific manual harvesting tool or the gripper of a robotized system, for example a sample changer or the cryostorage robotics of an automated crystal harvester. The identification pod combines human-readable and Datamatrix codes and can receive an RFID tag (§[Sec sec2.5]2.5). The NewPin puck is compatible with specific dewar slots that can also receive uni-puck, SPINEplus and miniSPINE pucks (Papp *et al.*, 2017 ▸). Up to 288 NewPins can be transported in a CX100 dewar using the eight-puck top-access canister or up to 252 using the seven-puck shelved canister (§[Sec sec2.7]2.7). A prototype with 66 pin slots was designed and the corresponding base was manufactured (Supplementary Fig. S9). From this prototype, a 64-slot beta version was designed and specified (not manufactured) to illustrate the ultimate capabilities of NewPin using test robotics. Nevertheless, it should be highlighted that the absence of individual sample wells keeps the crystals under liquid nitrogen only within a small puck-inclination range, thus making manual handling of the pucks delicate. This effect is nevertheless mitigated by the presence of the cover.

#### 2.3.4. NewPin manual handling tools   

Three manual handling tools have been developed: a pin-grabbing support (Fig. 6 ▸ *a*) that keeps the pin firmly and correctly oriented to facilitate mounting on the harvesting tool, a harvesting tool (Fig. 6 ▸ *b*) to hold a pin correctly during crystal harvesting, and a pin-extracting tool (Fig. 6 ▸ *d*) to remove a pin from a puck. The harvesting tool is a tweezer with a stud on each jaw that fits into the two holes on each sides of a pin. The harvesting process is similar to the process described above for mini­SPINE and is as follows: Step 1, the pin is inserted into the pin-grabbing support, gripped with the harvesting tool and is oriented manually at 45° to facilitate harvesting; Step 2, the crystal is harvested and cryoprotected; Step 3, the pin with its crystal is plunged into liquid nitrogen; Step 4, the pin is flipped by 135°, possibly using a flipping assistant similar to the miniSPINE puck-loading assistant (Fig. 4 ▸ *c*); Step 5, the pin with its crystal is inserted in a puck slot and released by pressing the button situated on the handle of the tool. To facilitate handling, the pins are locked in the harvesting and storing positions. As with miniSPINE, it is important to vitrify the crystal before flipping. This ensures faster cooling and secures the attachment of the crystal in the cryo-mount. Although manual harvesting is possible, NewPin is better adapted to fully robotic handling, in particular when considering the difficulties of visualizing puck slots in liquid nitrogen.

### 2.4. Pin orientation   

The precise orientation of the pins is particularly important with crystal mounts that set the crystal off the central pin axis, such as MicroMounts (MiTeGen) or in automated processes where the initial sample-holder orientation needs to be fixed, such as CrystalDirect (Zander *et al.*, 2016 ▸). Therefore, we have designed dedicated pin racks for each proposed sample-holder model (Supplementary Fig. S13) where the pins are pre-oriented. The specific pin slots for each sample-holder type include orienting locks for NewPin, holes with orientation flats for miniSPINE and orientation ridges for SPINEplus. It is also possible to fix the orientation of the SPINEplus pins upon manual mounting on a goniometer mount equipped with an orientation finger. It should be noted that pin orientation is intrinsic to the design of NewPin and must be respected upon handling.

### 2.5. Sample identification   

To facilitate sample tracking, different models of commercial sample holders and pucks have been proposed with optical identification tags such as barcodes, Datamatrix codes or coloured rings. The reliability of such optical identification methods often suffers from icing, surface degradation or fog, and is sensitive to lighting conditions. To overcome these issues, Rigaku Corporation (Tokyo, Japan) proposed and produced sample holders with custom RFID-tag identifiers. Nevertheless, their use remained limited and commercialization was stopped. Here, we propose to identify NewPin and miniSPINE sample holders with low-frequency RFID tags, possibly combined with Datamatrix codes. To identify pucks, we propose pods that can include a human readable code, a Datamatrix code and a high-frequency RFID tag.

The NewPin and miniSPINE radio-frequency (MSrf) sample holders (Figs. 3 ▸ *b* and 5 ▸ *a*) have been designed to receive RFID tags of 1.4 mm in diameter and 8 mm in length. A low-frequency glass tag was selected for reading though the body of the pins (in stainless steel), as well as when the pins are in sample-changer grippers. Standard tags from different manufacturers were tested. All showed a 30% frequency drift between room and liquid-nitrogen temperatures. To make them readable at both temperatures, the antennae of the tags were modified for a 15% frequency detuning against the nominal frequency at room temperature. This frequency adjustment allowed the use of a unique standard RFID reader antenna (tuned to 125 kHz) for both room and cryotemperature reading, simplifying future RFID reading stations. The major remaining problem with the RFID glass tags is a significant failure rate against temperature cycling between room and liquid-nitrogen temperatures. A partnership was therefore established with the company HID Global IDT (Granges-Veveyse, Switzerland) with the aim of reducing this failure rate to 0.1% after 100 cycles.

An identification pod for the NewPin puck (Fig. 6 ▸ *c*), miniSPINE puck (Fig. 4 ▸ *e*) and SPINEplus puck (Fig. 2 ▸ *c*) has been defined. The pod combines a human-readable ID and a Datamatrix label and can include a high-frequency RFID tag. Preliminary temperature-cycling tests on one batch of 50 commercially available high-frequency RFID tags showed a 2% failure rate over 100 cycles. Further improvements of the chips are required to reach a failure rate of 0.1% after 1000 cycles.

An important aspect of sample identification is the benefit-to-cost ratio. The total manufacturing cost of a sample holder is composed of three parts: the mechanical support, the crystal mount and the identifier. Unlike the widely used Datamatrix identification, the RFID tag-based identification proposed here represents a significant part of the total fabrication cost of a sample holder. On the other hand, the identification of a puck is much more affordable as it represents only a few percent of its total cost. Tracking projects with large numbers of equivalent crystals does not necessarily require the identification of each sample. Puck identification is most often sufficient. Similarly, fully automated environments can rely on the position of the sample holders in the pucks (Bowler *et al.*, 2015 ▸). Conversely, ligand-screening applications, where each crystal contains a different molecule, could benefit from sample-holder identification.

### 2.6. Pin length   

**Figure 7:**
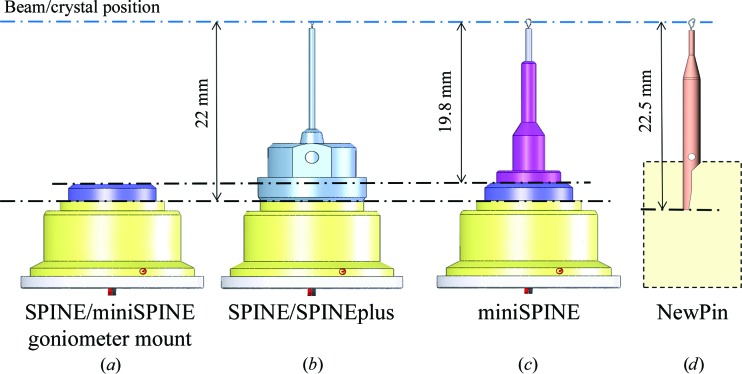
Sample-holder length compatibility. (*a*) Typical SPINE goniometer mount. (*b*) Positions of a SPINE/SPINEplus pin, (*c*) of a miniSPINE pin and (*d*) of a NewPin pin mounted on a goniometer socket.

Pin length is the usual way to name the length of the sample holder. At beamlines, it defines the distance from the gonio­meter mount to the X-ray beam. The SPINE sample-holder standard has a fixed pin length of 22 mm (from the base of the cap to the crystal or beam position). Adopting a unique pin length significantly reduced the beamline-compatibility issues and facilitated the use of kappa goniometers presenting limited tolerance against pin-length variations (Brockhauser *et al.*, 2013 ▸). Particular care was taken in the choice of the NewPin and miniSPINE sample-holder lengths in order to facilitate the design of compatible goniometer mounts (Fig. 7 ▸).

### 2.7. Puck-handling tools and dewar canisters   

**Figure 8:**
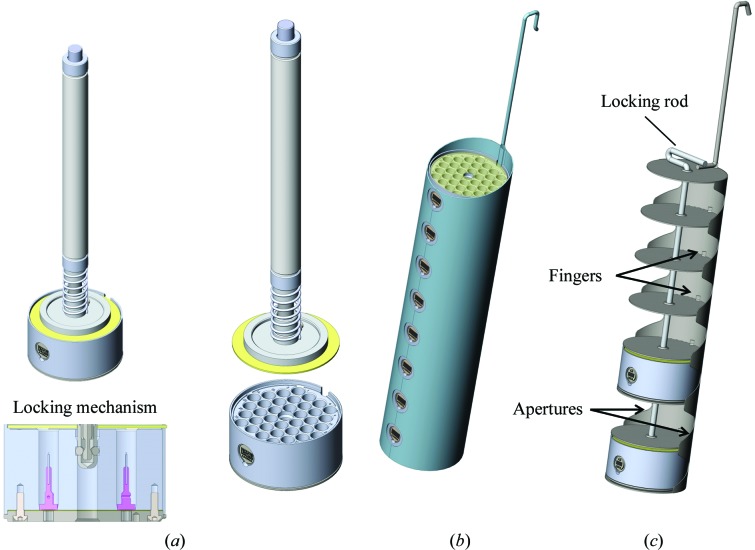
Puck-handling tool and canisters. (*a*) Puck-handling tool grabbing a puck with its cover and releasing the puck while keeping the cover, also showing a cut view of the locking mechanism. (*b*) Eight-puck top-access canister. (*c*) Seven-puck shelved canister with locking rod, antirotation fingers and apertures for manual gripper.

A common tool (Fig. 8 ▸ *a*, Supplementary Fig. S10) was developed to manipulate the NewPin, miniSPINE and SPINEplus pucks. Three spring-loaded balls at the tip of the tool grab the puck from a groove when the tool is inserted into the centre of the puck. The puck can be released with or without the cover by pressing the button at the top of the tool. When the puck is held in the tool, a spring pushes a disc against the top side of the puck. The friction between this disc and the puck allows the puck to be aligned with an orientation finger in a sample-changer dewar slot. Additionally, two canister designs are proposed for transport dewars. In the first design, the eight-puck top-access canister (Fig. 8 ▸ *b*), the pucks are simply stacked and are directly accessible using a tool similar to the puck-handling tool described above, but with an extended handle. In this case, the pucks are accessible in first-in-last-out (FILO) order. The second model, the seven-puck shelved canister (Fig. 8 ▸ *c*, Supplementary Fig. S10) provides random puck access but requires an additional tool to handle the pucks. Orientation fingers keep the pucks positioned with identification pods accessible for reading. The pucks can then be locked in position with a rod.

## 3. Testing   

Manual handling of the SPINEplus, miniSPINE and NewPin sample holders as well as of the related manipulation tools has been tested during the development phase. Once selected for the first implementation of a future sample-holder standard, the miniSPINE system was tested at 12 different partner sites using evaluation kits (Supplementary Fig. S5). Its design was then upgraded following the feedback received. In parallel, all of the models have been tested under real conditions (crystal harvesting, automated sample mounting and data collection) at the ESRF–EMBL–India BM14 beamline using a FlexED8 sample changer (Papp *et al.*, 2017 ▸).

## 4. Results   

Here, we only report on the manual usability of the sample holders. The results related to robotic sample transfers are published in the accompanying article (Papp *et al.*, 2017 ▸) as they depend on the robot grippers, the transfer times and the precision of the robotics used. The SPINEplus sample holders were found to be easy to manipulate as they are identical in size to the sample holders commonly used at beamlines. The miniSPINE harvesting tool was judged to be convenient. However, owing to the angle between the pin and the tool, some users found it more difficult to use than the usual straight tools that are used to handle the SPINE sample holders where the orientation of the loop can be easily selected. A major problem was encountered with NewPin and miniSPINE pucks when inserting the pins with a crystal mounted on them into a puck under liquid nitrogen. Owing to the small size of the wells, it can be difficult to know whether a position is occupied or free. An additional difficulty when using NewPin was to properly orient the pins to plug them correctly into the puck slots. This made it clear that NewPin should be reserved for fully robotic systems, including the crystal-harvesting step. For miniSPINE, the puck-loading assistant (Fig. 4 ▸ *c*) greatly improved the manual harvesting process (Supplementary Video S1).

## 5. Discussion   

Two new sample holders for macromolecular cryocrystallography have been developed together with corresponding pucks and robotic and manual handling tools to enhance crystal-processing times at beamlines and to reduce the handling effort and transportation costs. miniSPINE allows the storage of 36 samples in a puck. NewPin offers a storage density of up to 64 samples per puck combined with highly accurate positioning on goniometers. The two models can be handled with the same robot gripper and use pucks with the same footprint. They fit in a specific uni-puck-compliant dewar slot and in canisters compatible with CX100 shipping dewars. A goniometer mount compatible with SPINE and miniSPINE was also developed to facilitate the integration of miniSPINE at beamlines. The NewPin model requires a specific gonio­meter mount and is more difficult to handle manually. It is adapted to fully automated pipelines covering all of the steps from crystal harvesting to processing at beamlines. The maximum storage density of the NewPin pins could potentially exceed 64 pins per puck as it depends on the space allocated around the pins for handling. This is related to the outer dimensions of the gripper and to the precision of the handling robotics. Finally, a modified SPINE sample holder called SPINEplus, which is backwards-compatible with SPINE, was developed together with a miniSPINE/NewPin dewar slot compliant puck. This interoperable line of sample holders and pucks should facilitate the transition from the current SPINE sample-holder standard to miniSPINE and further to NewPin, in particular on beamlines that are equipped with flexible sample changers based on six-axis industrial robots and tool changers. Initially, sample tracking was identified as highly important when moving to the densities proposed here; therefore, both pins and pucks are ready to receive RFID tags. Work is continuing to improve the reliability of the RFID tags used to identify the pins so that they can resist the extreme thermal cycling experienced during their lifetime. The identification of the pucks also includes Datamatrix and human-readable labels. Defined as an important feature at the beginning of the project, the identification of the pins was over time judged to be less important than the identification of pucks. In practice, even when available, SPINE-standard pin identification is rarely used, probably because of the additional effort needed during crystal harvesting. Currently, sample tracking almost exclusively relies on the position of the pins in human-identifiable pucks. Furthermore, as automation becomes more extensive, the potential for human error decreases. In the near future, projects where individual sample tracking is important should benefit from automated harvesting and storing, making pin identification unnecessary.

The first pin and puck prototypes manufactured demonstrated that the main drawback of increasing density is the difficulty in manually handling the pins. A number of tools and assistants were developed to facilitate crystal harvesting and the storage of the pins in the pucks. The feedback received from partner institutes and companies on the miniSPINE manual handling tools were integrated into the present design. Nevertheless, some limitations remain with the harvesting tool, where the pins being set at 45° (Figs. 2 ▸, 4 ▸ and 6 ▸) can reduce the ability of the user to see and manipulate the crystals in trays during harvesting. We should however point out that the main goal of this work is to propose new sample holders and that all of the related devices have essentially been developed to assess the possibility of their manual handling. As with existing sample holders, further tools can develop over time. The second important aspect considered was integration at beamlines. The main reason for miniSPINE being proposed for the initial implementation of a new sample-holder standard is the potential to operate a beamline with both SPINE and miniSPINE, and because manual handling is easier than with NewPin. NewPin is therefore considered as a second implementation phase, or for a highly demanding fully integrated platform from crystallization to data collection. While sample turnover could be dramatically increased at most recent third-generation synchrotron beamlines, new data-collection methods such as *in situ* data collection and serial crystallography (Bingel-Erlenmeyer *et al.*, 2011 ▸; Axford *et al.*, 2012 ▸; Gati *et al.*, 2014 ▸; Zander *et al.*, 2015 ▸) disrupt the relationship between crystal and sample holder, making future requirements unclear. Similarly, the emergence of XFELs and the upgrade plans of many synchrotrons worldwide are making sample delivery more and more diversified (Lyubimov *et al.*, 2015 ▸; Oghbaey *et al.*, 2016 ▸; Sugahara *et al.*, 2015 ▸; Weierstall *et al.*, 2014 ▸), and therefore the requirements for future sample holders are even less predictable. Nevertheless, the new sample holders proposed here should reduce the overall sample-handling efforts and costs, as well as accelerating the alignment of crystals or areas to scan at beamlines. This will be particularly true when they are connected to future automated harvesting systems that are anticipated to register crystal coordinates with individual pins.

Medium-size batches of the different sample holders and the associated pucks have been manufactured for testing and attempts have been made to find manufacturers able to produce them in large quantities and at affordable cost. Key companies working in the field of consumables for MX have been associated with the project at an early stage to prepare for the commercialization of the hardware involved. For large production batches, the end price of the pins without RFID tags should be comparable to the price of the current pins (batches larger than 5000 units) and the end price of the pucks expressed in cost per pin stored should be lower than the average price of current pucks (batches larger than 100 units). Moving from this feasibility study to a widely approved standard is now the next important step. Knowing that the production costs are largely dependent on the quantity produced, and for NewPin on the investments made in tooling, one of the main difficulties is to have affordable consumables available for the pilot test sites. At the time of writing, beamlines are hesitant to upgrade their robotics before consumables are available, especially as many synchrotron sites have already invested in large-capacity dewars to cope with increasing sample turnover, currently making sample-storage density a less critical issue. Therefore, the deployment of miniSPINE will start at a limited number of, mostly European, pilot beamlines, in parallel with the production of the first batches of consumables.

The new sample supports presented here represent a new opportunity for MX experiments where automation is playing an ever-increasing role in seeking high reproducibility and high throughput. It is hoped that miniSPINE, and eventually NewPin, will play a central part in the future of ‘gene to structure’ and in more sophisticated drug-development pipelines.

## Supplementary Material

Technical specifications of miniSPINE sample holders and pucks.. DOI: 10.1107/S2059798317013742/gm5053sup1.pdf

Technical specifications of NewPin sample holders and pucks.. DOI: 10.1107/S2059798317013742/gm5053sup2.pdf

Technical specifications of SPINEplus sample holders and pucks.. DOI: 10.1107/S2059798317013742/gm5053sup3.pdf

Supplementary Figures.. DOI: 10.1107/S2059798317013742/gm5053sup4.pdf

Video of manual harvesting process using miniSPINE sample holder and corresponding manual tools.. DOI: 10.1107/S2059798317013742/gm5053sup5.wmv
