# Supplementary material for: Towards a compact and precise sample holder for macromolecular crystallography
Source: Acta Crystallogr D Struct Biol. 2017 Sep 29;73(Pt 10):829–40. doi: 10.1107/S2059798317013742 (PMC5633908; doi:10.1107/S2059798317013742)
Supplement: Supplementary file 1 [file d-73-00829-sup1.pdf]

Acta Cryst D

STRUCTURAL
BIOLOGY

Volume 73 (2017)

Supporting information for article:

Towards a compact and precise sample holder for macromolecular crystallography

Gergely Papp, Christopher Rossi, Robert Janocha, Clement Sorez, Marcos Lopez-Marrero, Anthony Astruc, Andrew McCarthy, Hassan Belrhali, Matthew W. Bowler and Florent Cipriani

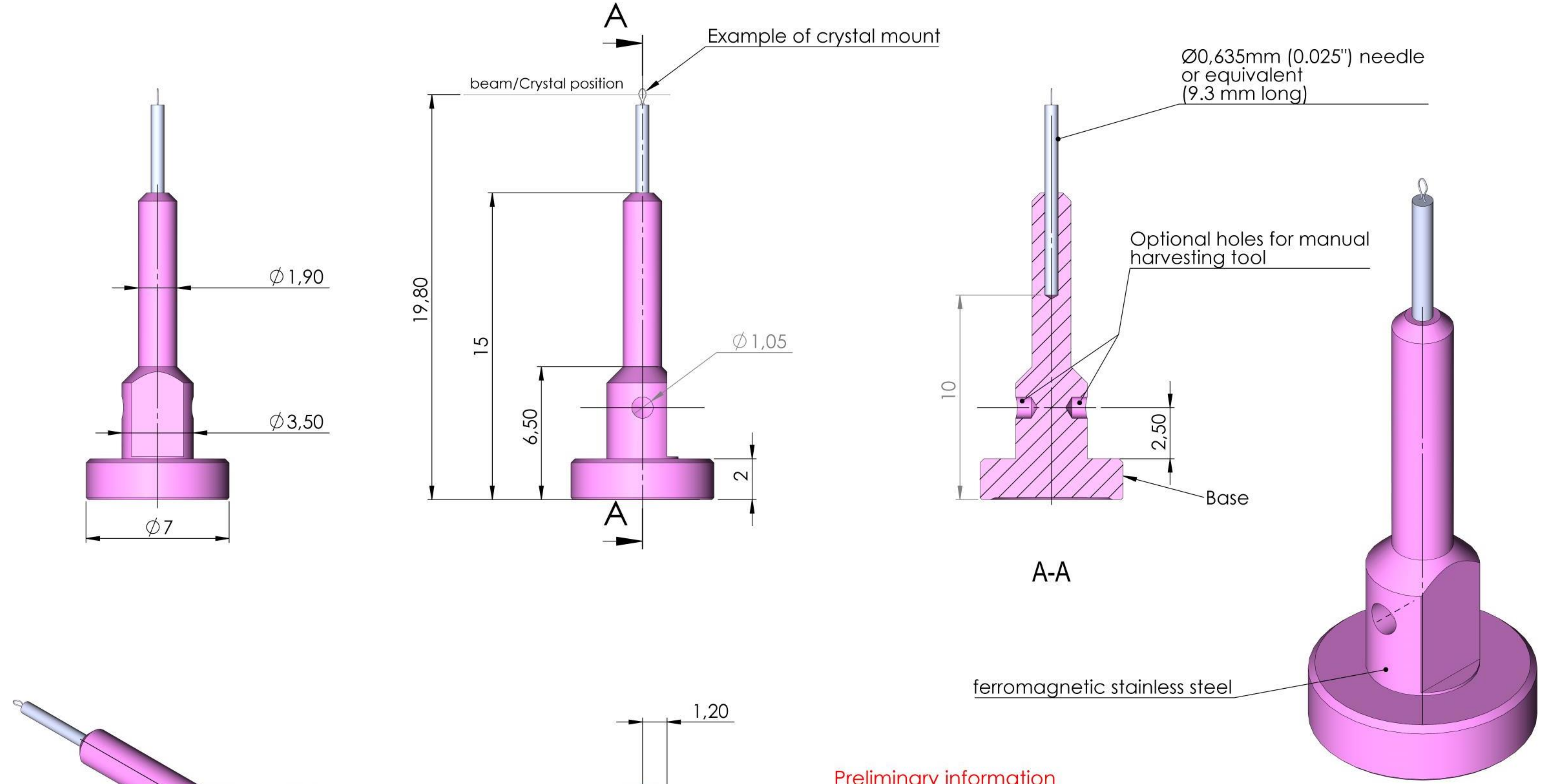

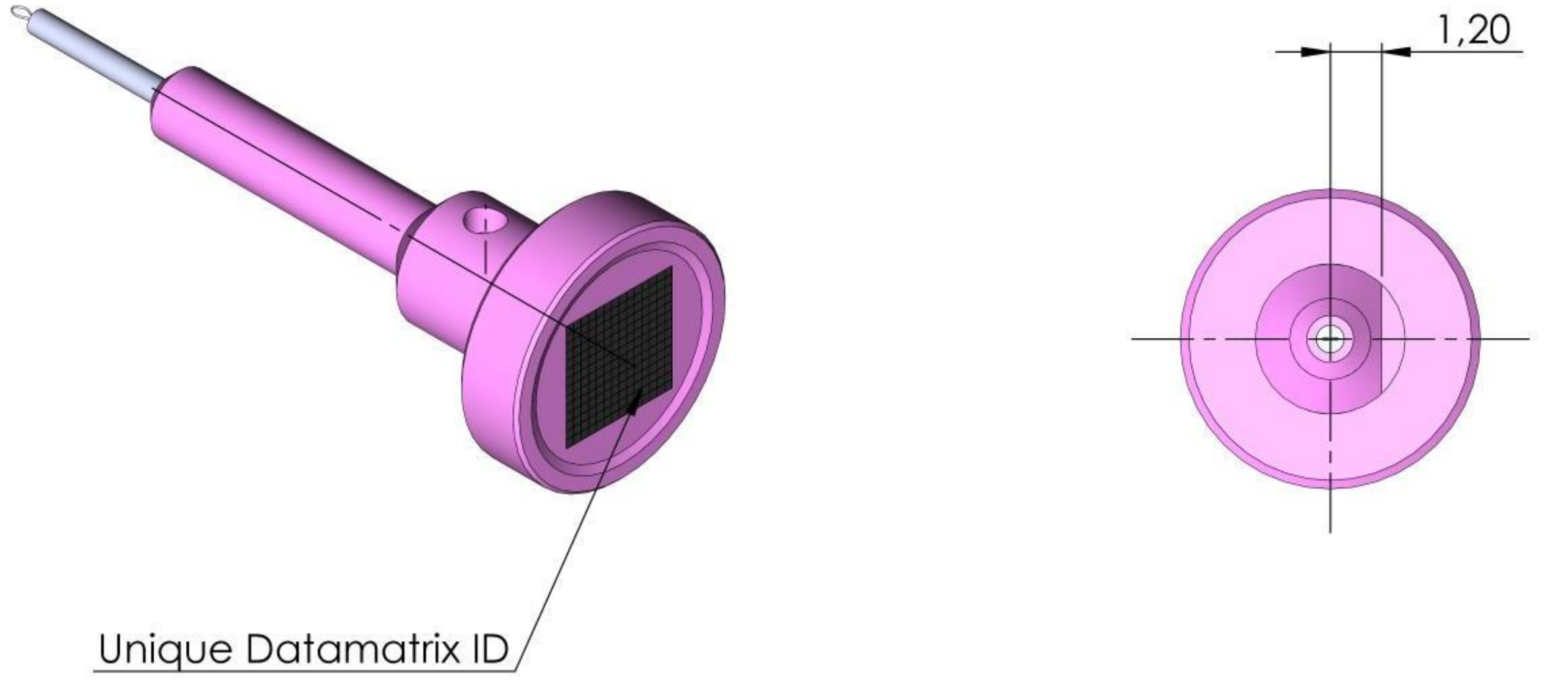

Preliminary information

	Date	Modification	Auteur
c	24/10/2016		C.ROSSI
b	27/07/2016		C.ROSSI
a	07/06/2016		C.ROSSI

Titre
miniSPINE sample holder V6 (base+needle)

Ce dessin est la propriété exclusive de EMBL toute reproduction ou utilisation de l'objet représenté sont interdits sans autorisation

Numéro - Indice de mise à jour **1120 70-C**	Projet : NewPin	
	Sous-ensemble : miniSPINE sample holder	
Matière : -	Remarque :	Qté :
Dessiné : C.ROSSI	Date : 26/05/2016	Echelle : 5:1

EMBL
Antenne de Grenoble
Laboratoire Européen de Biologie Moléculaire
71av des Martyrs - 38000 Grenoble
téléphone 0 476 207 188 - fax 0 476 207 199

Format A3

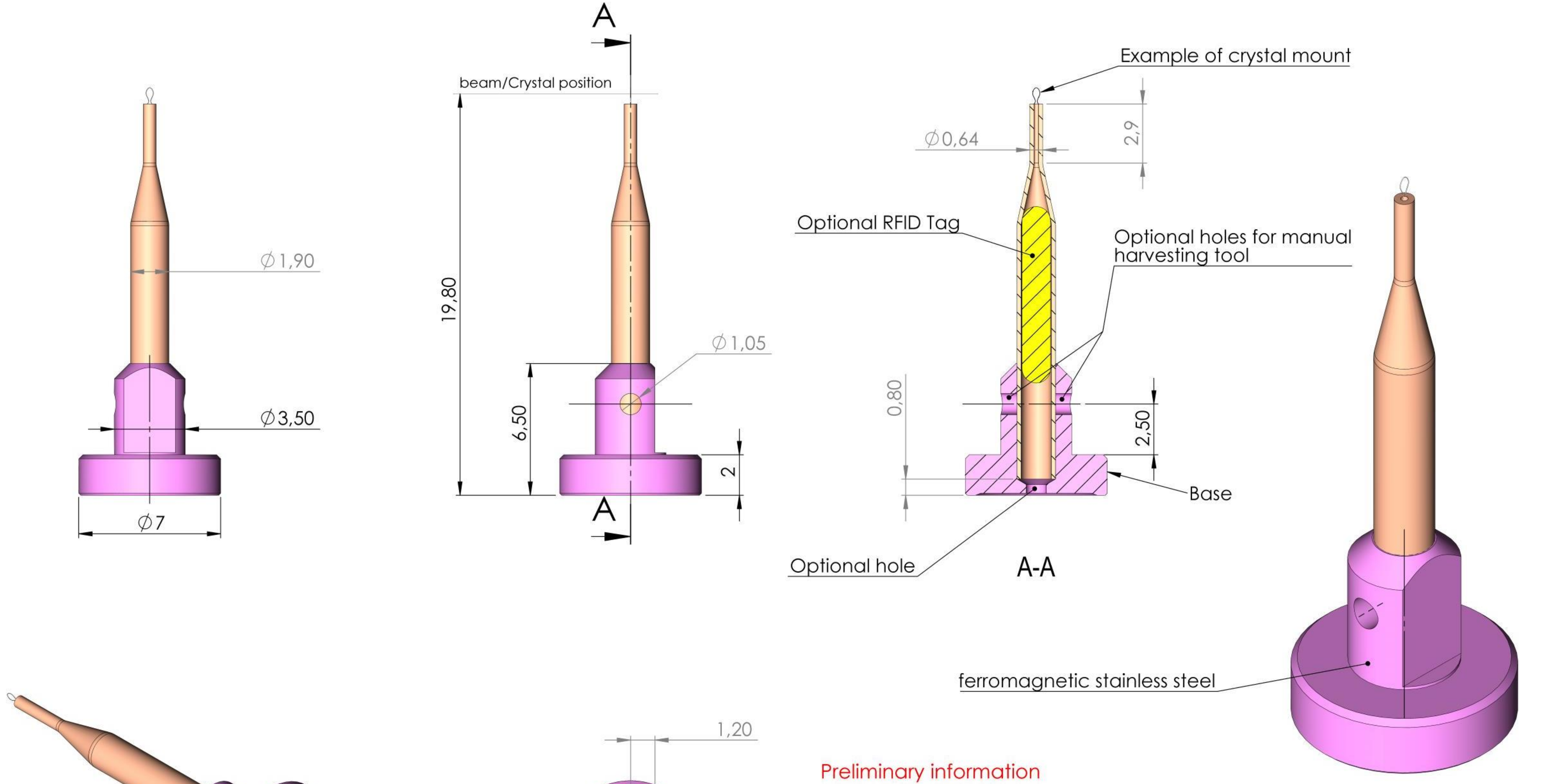

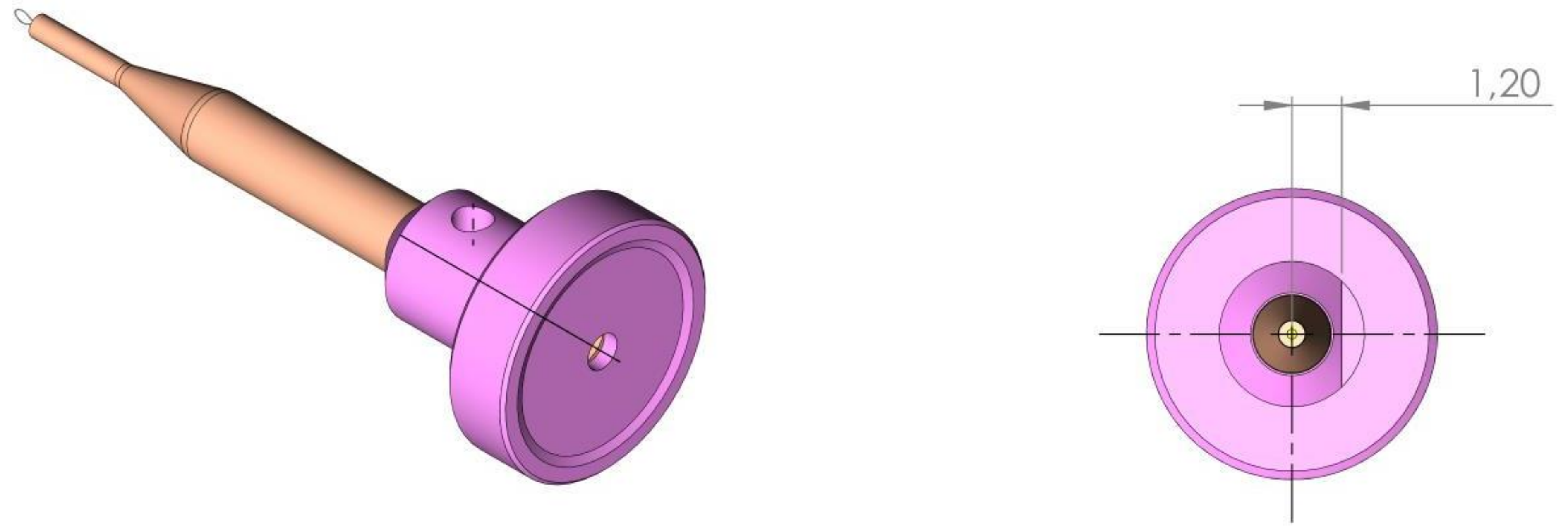

Preliminary information

c			
b			
a			
	Date	Modification	Auteur

Titre

miniSPINErf sample holder Beta1

Ce dessin est la propriété exclusive de EMBL toute reproduction ou utilisation de l'objet représenté sont interdits sans autorisation

Numéro - Indice de mise à jour **1120 90**	Projet : NewPin<br>Sous-ensemble : miniSPINE sample holder	
Matière : -	Remarque :	Qté :
Dessiné : C.ROSSI	Date : 17/03/2017	Echelle : 5:1

EMBL
Antenne de Grenoble
Laboratoire Européen de Biologie Moléculaire
71av des Martyrs - 38000 Grenoble
téléphone 0 476 207 188 - fax 0 476 207 199

Format A3

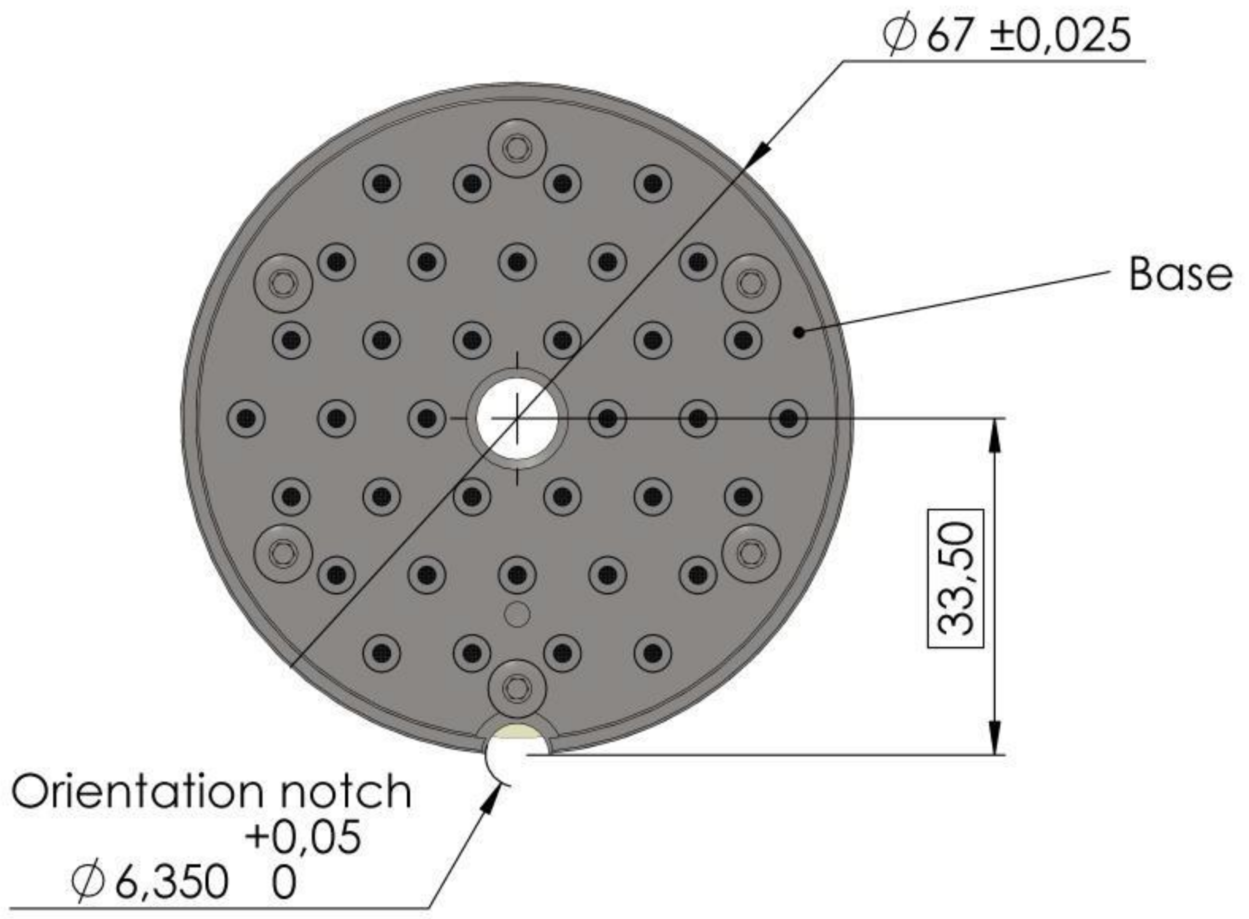

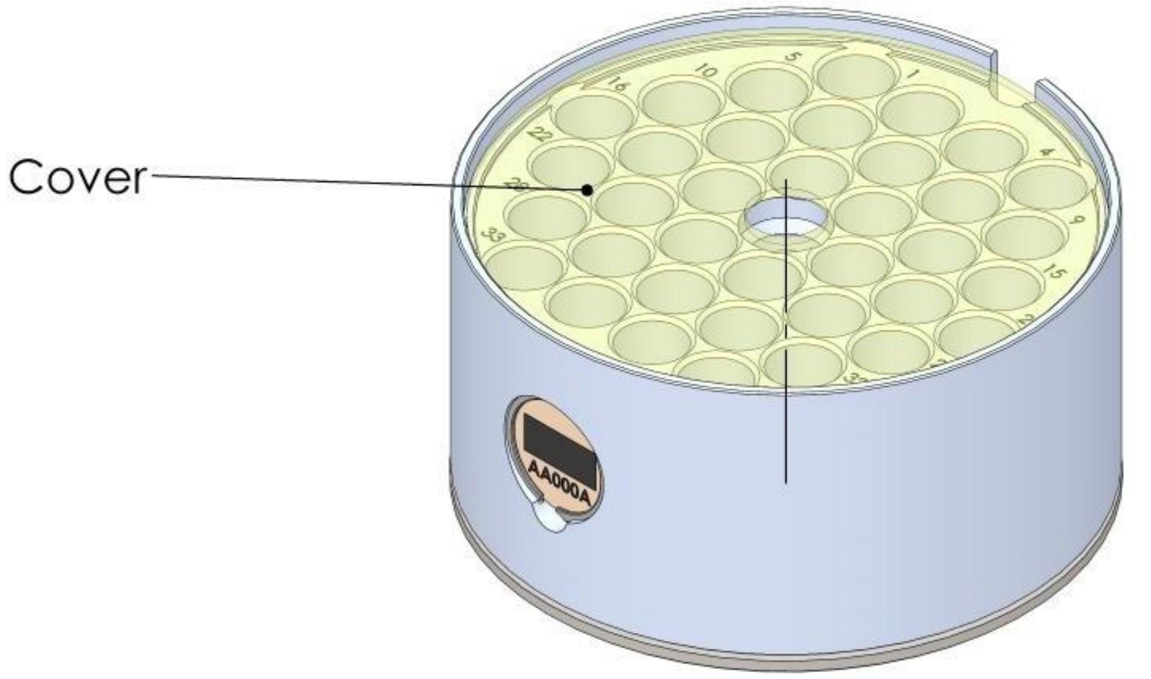

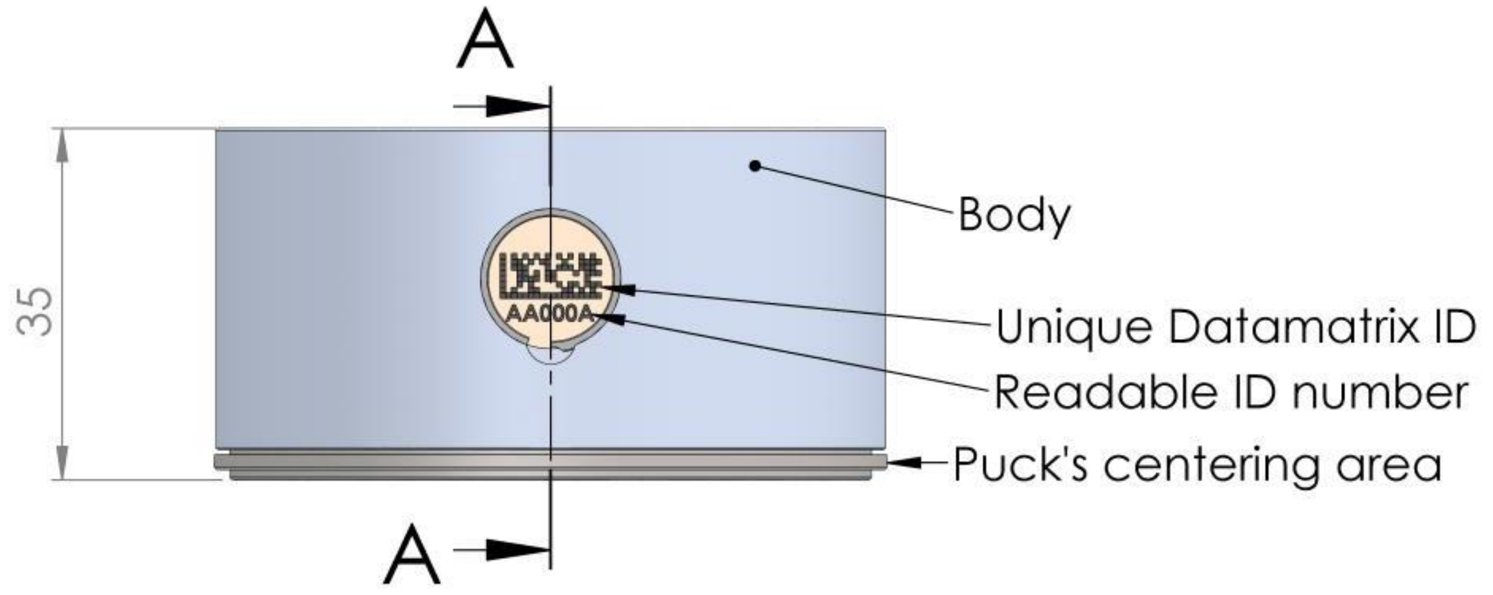

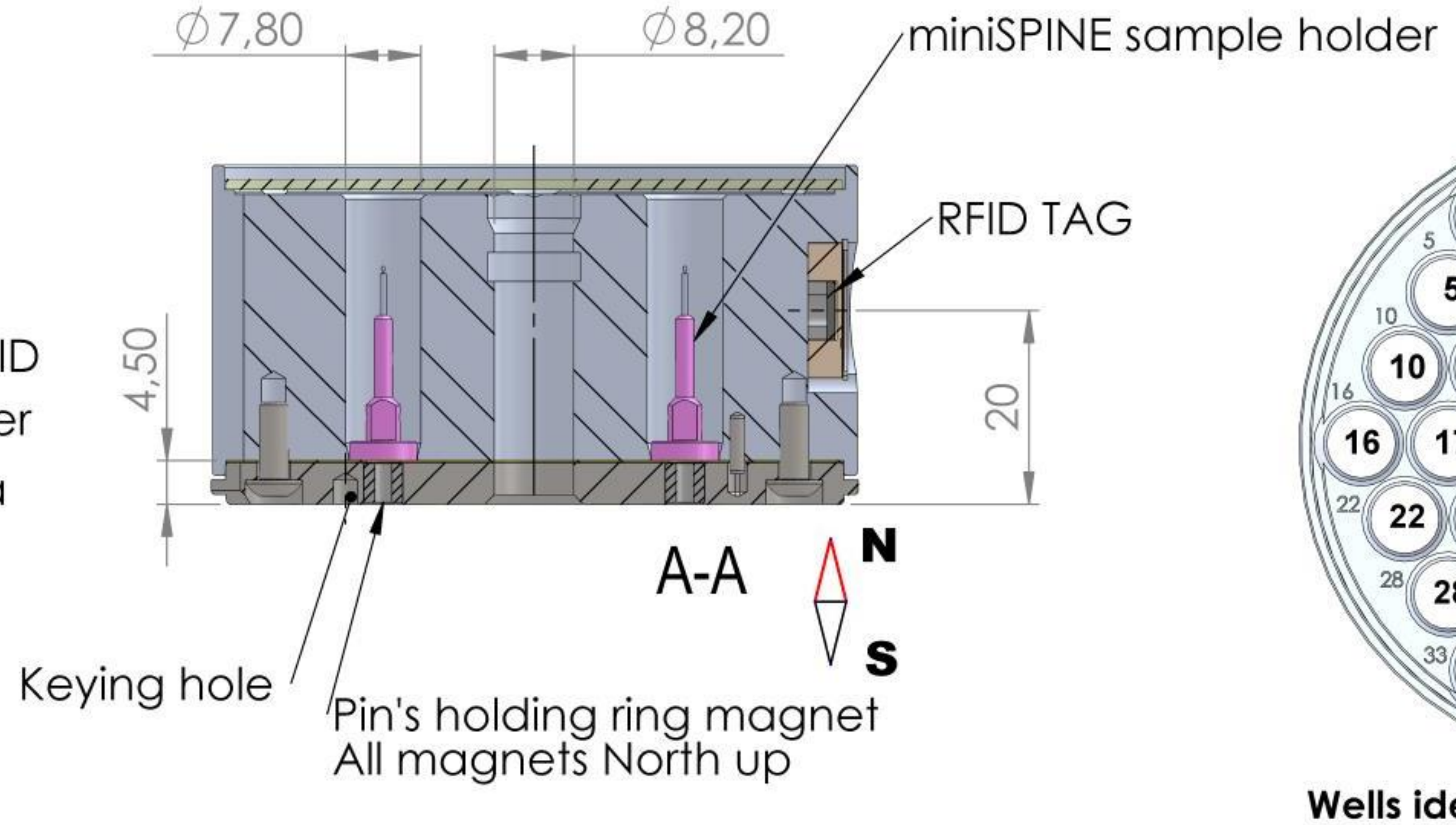

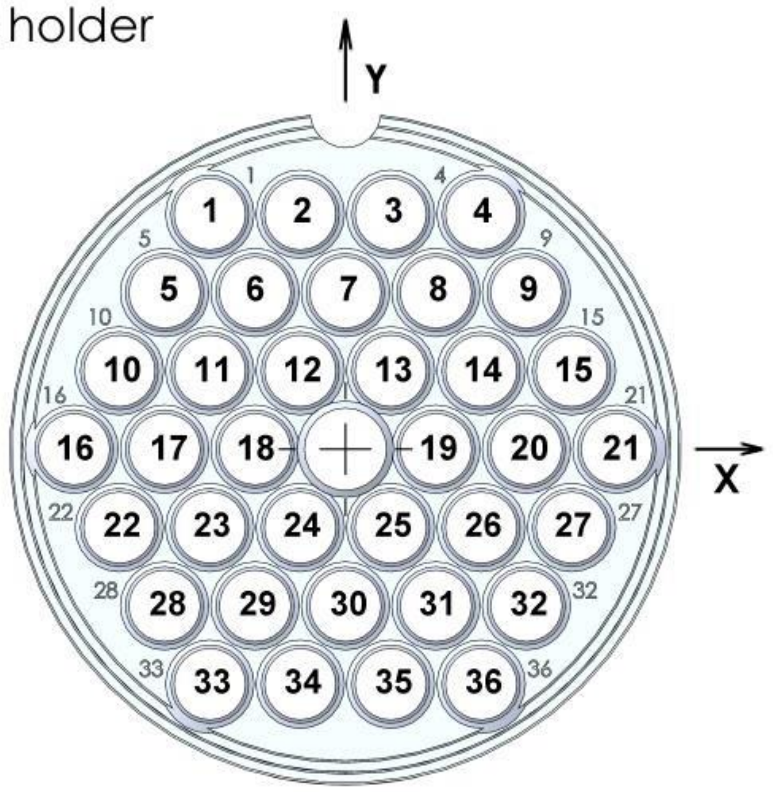

Wells identification and coordinates

36 wells arranged as an hexagonal matrix of 9x9

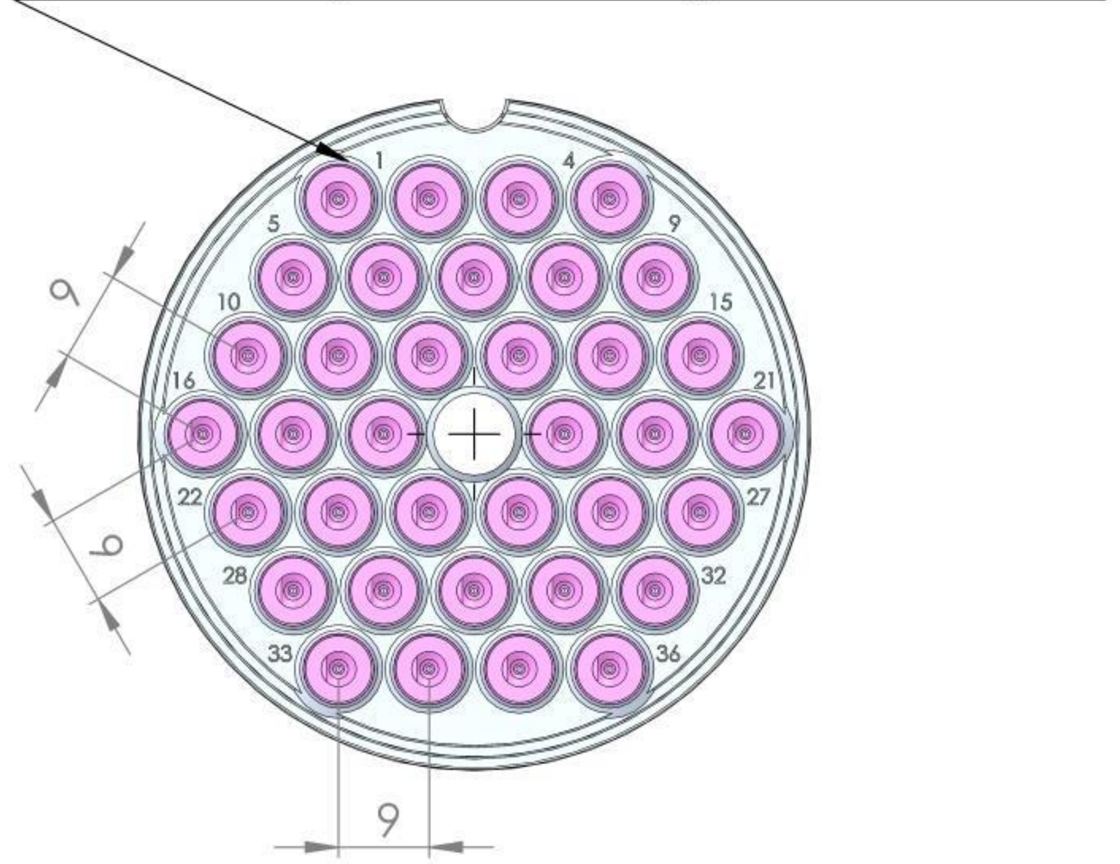

Well	X	Y
1	-13,5	23,38
2	-4,5	23,38
3	4,5	23,38
4	13,5	23,38
5	-18	15,59
6	-9	15,59
7	0	15,59
8	9	15,59
9	18	15,59
10	-22,5	7,79
11	-13,5	7,79
12	-4,5	7,79
13	4,5	7,79
14	13,5	7,79
15	22,5	7,79
16	-27	0
17	-18	0
18	-9	0
19	9	0
20	18	0
21	27	0
22	-22,5	-7,79
23	-13,5	-7,79
24	-4,5	-7,79
25	4,5	-7,79
26	13,5	-7,79
27	22,5	-7,79
28	-18	-15,59
29	-9	-15,59
30	0	-15,59
31	9	-15,59
32	18	-15,59
33	-13,5	-23,38
34	-4,5	-23,38
35	4,5	-23,38
36	13,5	-23,38

Preliminary informations

	Date	Modification	Auteur
d	22/08/2016	new tag holder, higher magnets	C.ROSSI
c	25/07/2016		C.ROSSI
b			C.ROSSI

Titre

miniSPINE Puck V3

Ce dessin est la propriété exclusive de EMBL toute reproduction ou utilisation de l'objet représenté sont interdits sans autorisation

Numéro - Indice de mise à jour
1122 01 00-D

Projet : NewPin

Sous-ensemble : miniSPINE puck

Matière :

Remarque : Qté : 1

Dessiné : C.ROSSI

Date : 11/01/2016

Echelle : 1:1

EMBL
Antenne de Grenoble
Laboratoire Européen de Biologie Moléculaire
71av des Martyrs - 38000 Grenoble
téléphone 0 476 207 188 - fax 0 476 207 199

Format A3
