# Supplementary material for: Towards a compact and precise sample holder for macromolecular crystallography
Source: Acta Crystallogr D Struct Biol. 2017 Sep 29;73(Pt 10):829–40. doi: 10.1107/S2059798317013742 (PMC5633908; doi:10.1107/S2059798317013742)
Supplement: Supplementary file 2 [file d-73-00829-sup2.pdf]

STRUCTURAL
BIOLOGY

Volume 73 (2017)

Supporting information for article:

Towards a compact and precise sample holder for macromolecular crystallography

Gergely Papp, Christopher Rossi, Robert Janocha, Clement Sorez, Marcos Lopez-Marrero, Anthony Astruc, Andrew McCarthy, Hassan Belrhali, Matthew W. Bowler and Florent Cipriani

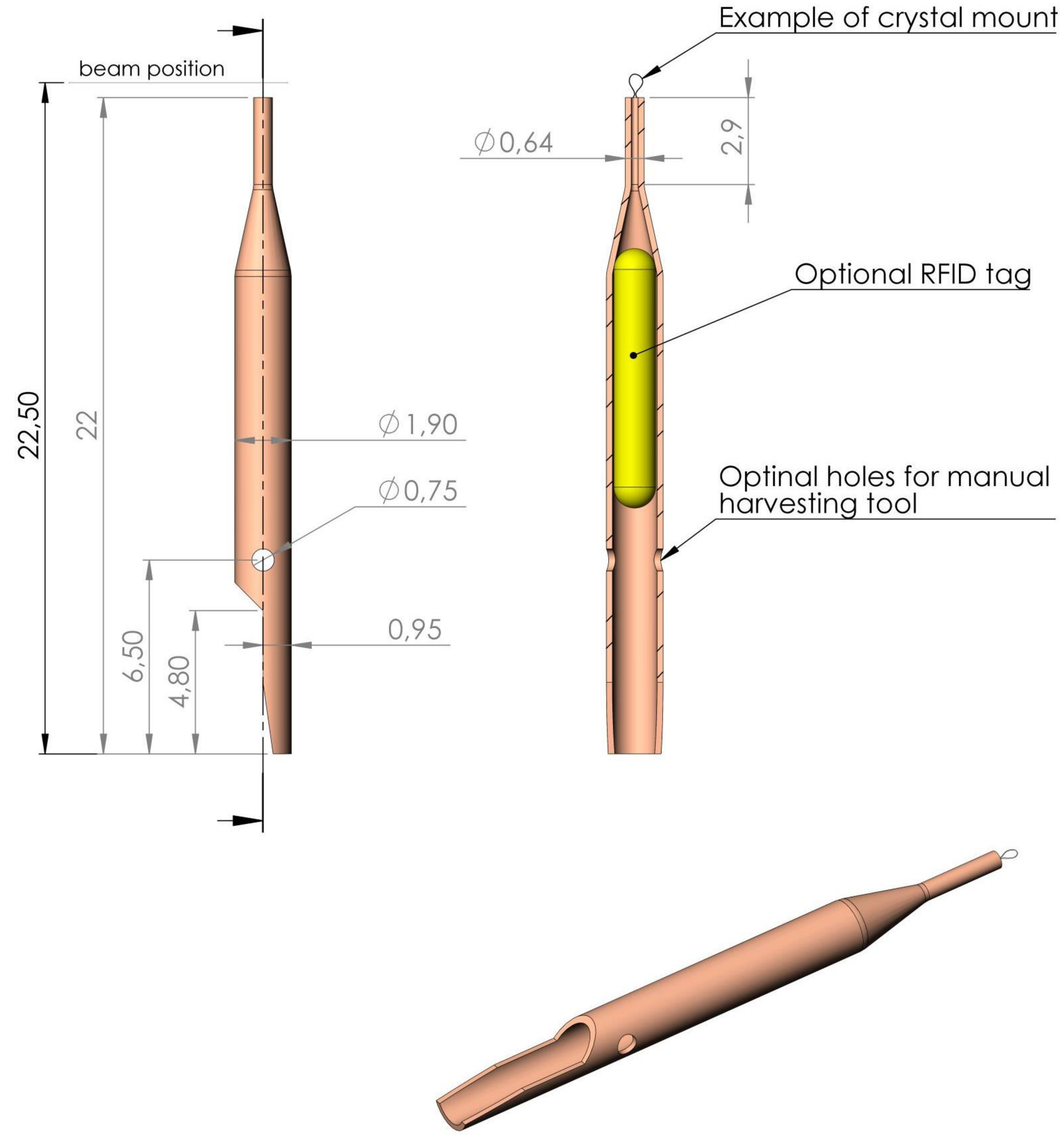

Preliminary information

	Date	Modification	Auteur
c			
b			
a			

Titre

NewPin sample holder V7

(NewPin-ID150-XX-V7)

Ce dessin est la propriété exclusive de EMBL toute reproduction ou utilisation de l'objet représenté sont interdits sans autorisation

Numéro - Indice de mise à jour **1110 000**	Projet : NewPin		
	Sous-ensemble : NewPin sample holder		
Matière :	Remarque :	Qté :	
Dessiné : C.ROSSI	Date : 12/12/2016	Echelle : 5:1	

EMBL
Antenne de Grenoble
Laboratoire Européen de Biologie Moléculaire
71 av. des martyrs - 38000 Grenoble
téléphone 0 476 207 188 - fax 0 476 207 199

Format A4

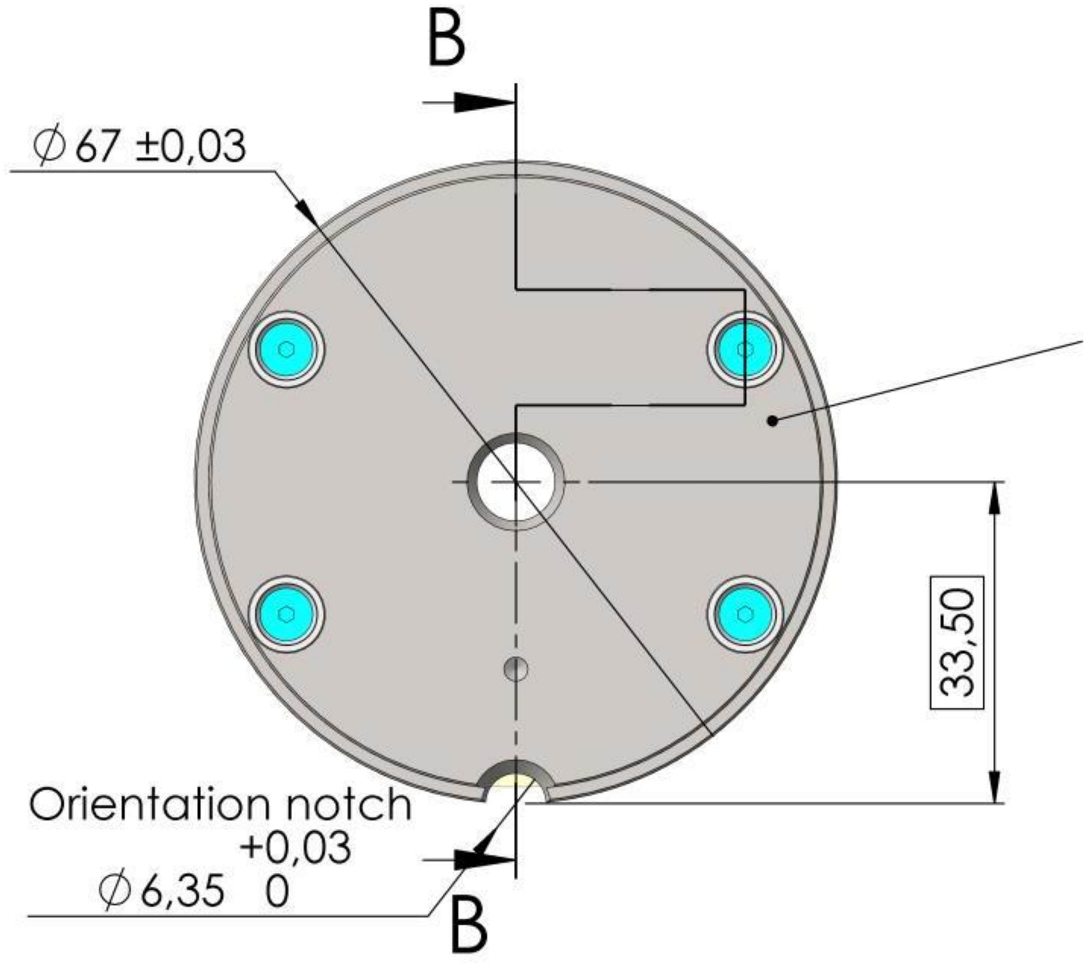

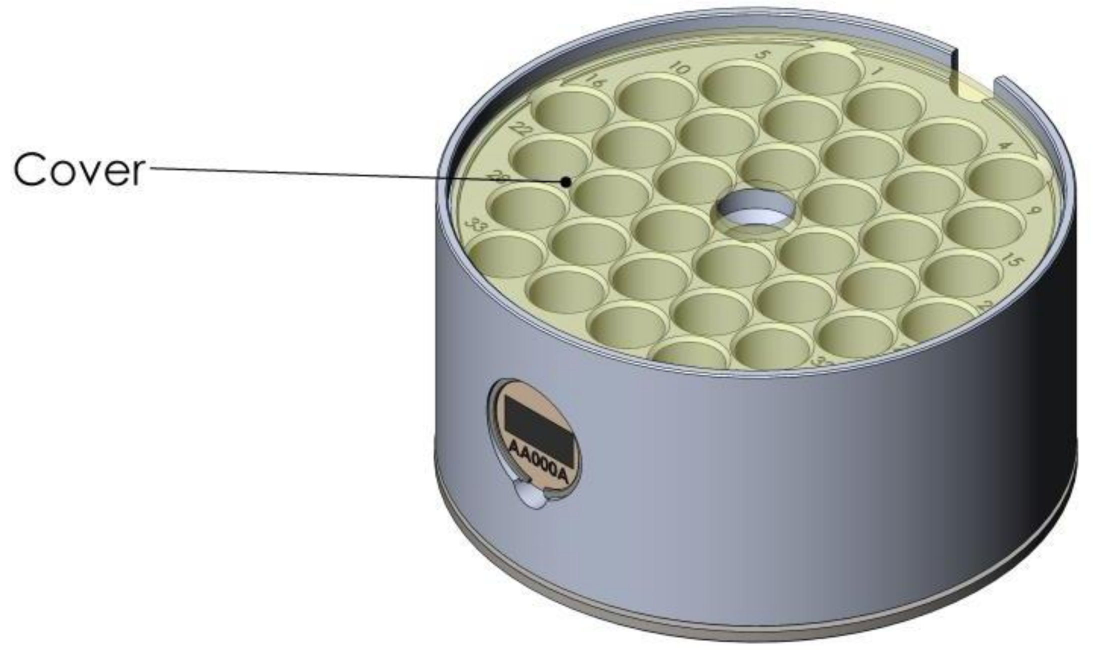

Well	X	Y
1	-13,5	23,38
2	-4,5	23,38
3	4,5	23,38
4	13,5	23,38
5	-18	15,59
6	-9	15,59
7	0	15,59
8	9	15,59
9	18	15,59
10	-22,5	7,79
11	-13,5	7,79
12	-4,5	7,79
13	4,5	7,79
14	13,5	7,79
15	22,5	7,79
16	-27	0
17	-18	0
18	-9	0
19	9	0
20	18	0
21	27	0
22	-22,5	-7,79
23	-13,5	-7,79
24	-4,5	-7,79
25	4,5	-7,79
26	13,5	-7,79
27	22,5	-7,79
28	-18	-15,59
29	-9	-15,59
30	0	-15,59
31	9	-15,59
32	18	-15,59
33	-13,5	-23,38
34	-4,5	-23,38
35	4,5	-23,38
36	13,5	-23,38

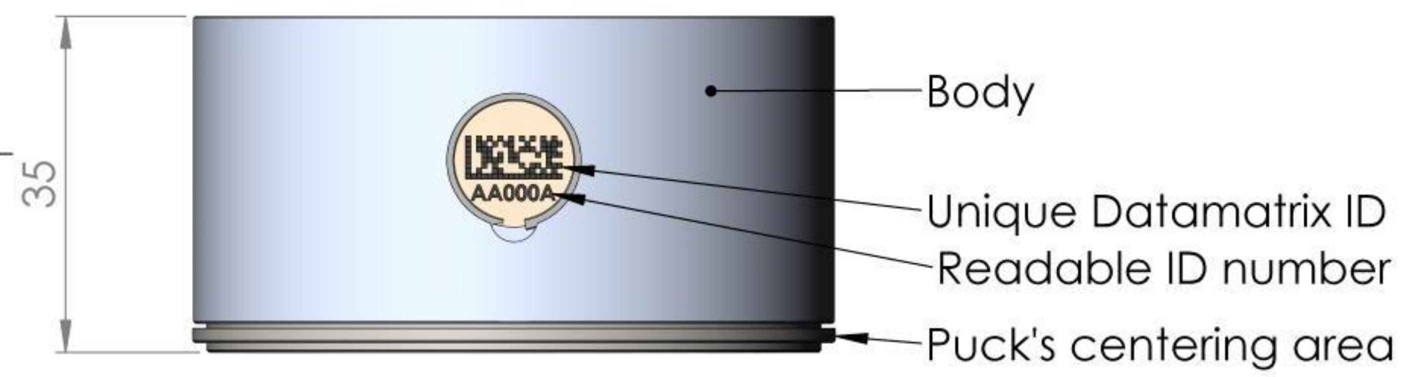

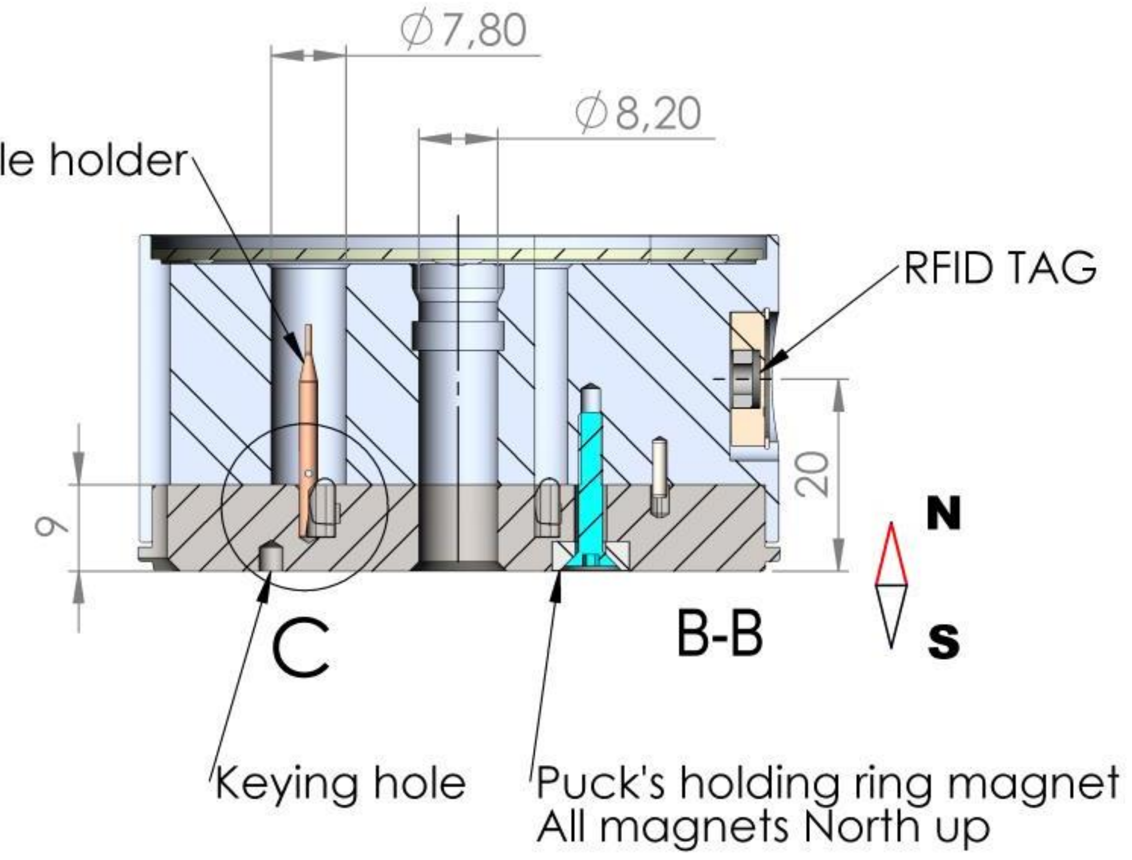

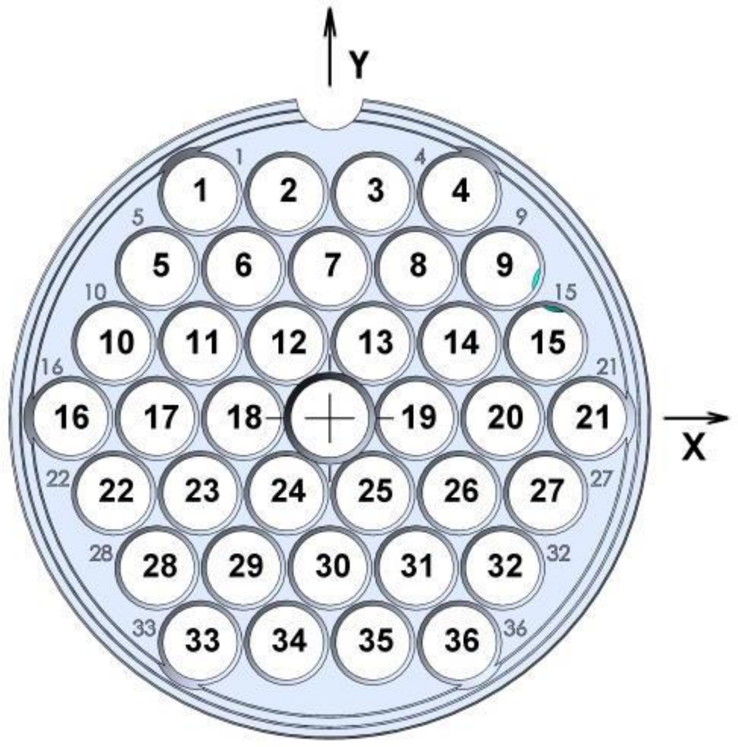

Wells identification and coordinates (coordinates of the pins)

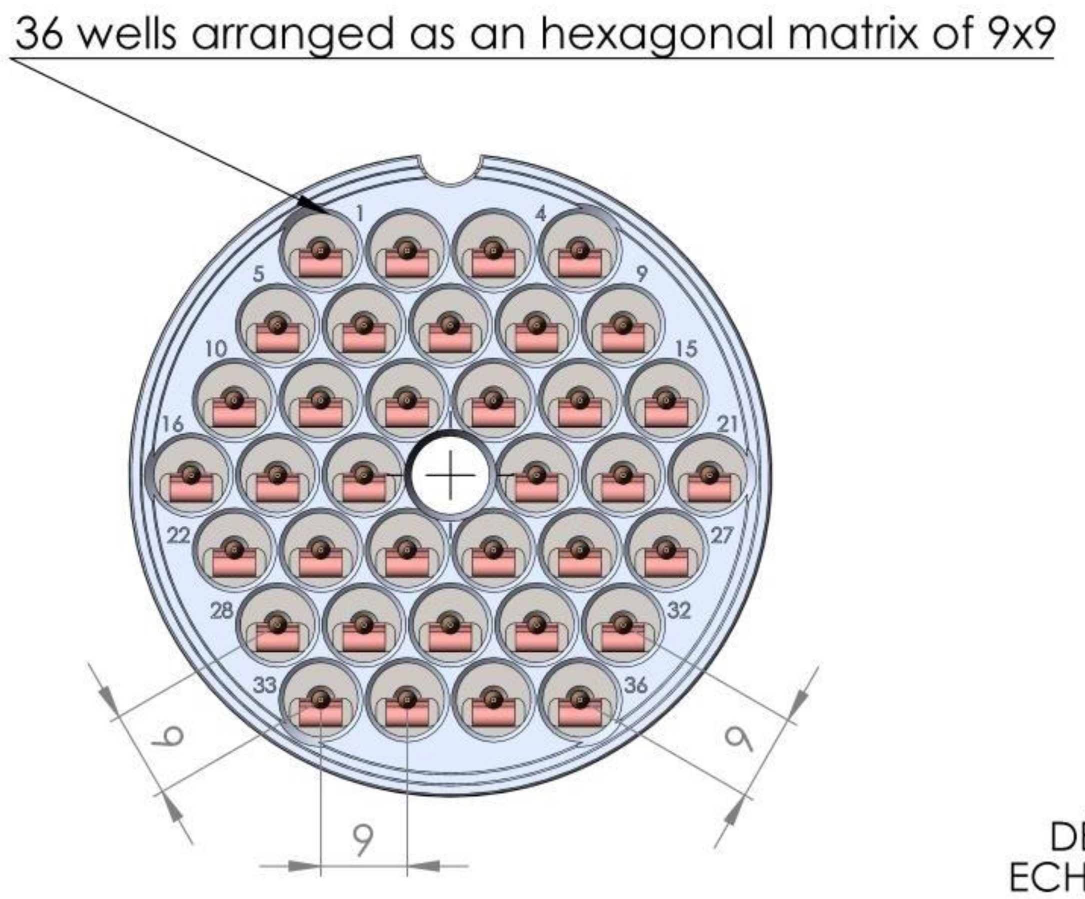

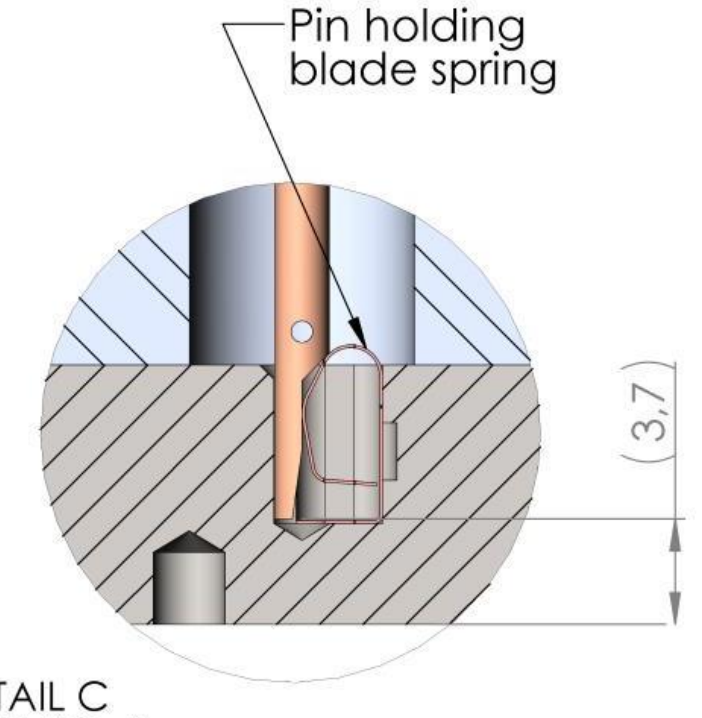

DÉTAIL C
ECHELLE 3 : 1

Preliminary information

	Date	Modification	Auteur
c			
b			
a			

Titre

NewPin puck V1

Ce dessin est la propriété exclusive de EMBL toute reproduction ou utilisation de l'objet représenté sont interdits sans autorisation

Numéro - Indice de mise à jour **11121 000**	Projet : NewPin		
	Sous-ensemble : NewPin puck		
Matière :	Remarque :	Qté :	
Dessiné : C.ROSSI	Date : 07/12/2016	Echelle : 1:1	

EMBL
Antenne de Grenoble
Laboratoire Européen de Biologie Moléculaire
71av des Martyrs - 38000 Grenoble
téléphone 0 476 207 188 - fax 0 476 207 199

Format A3

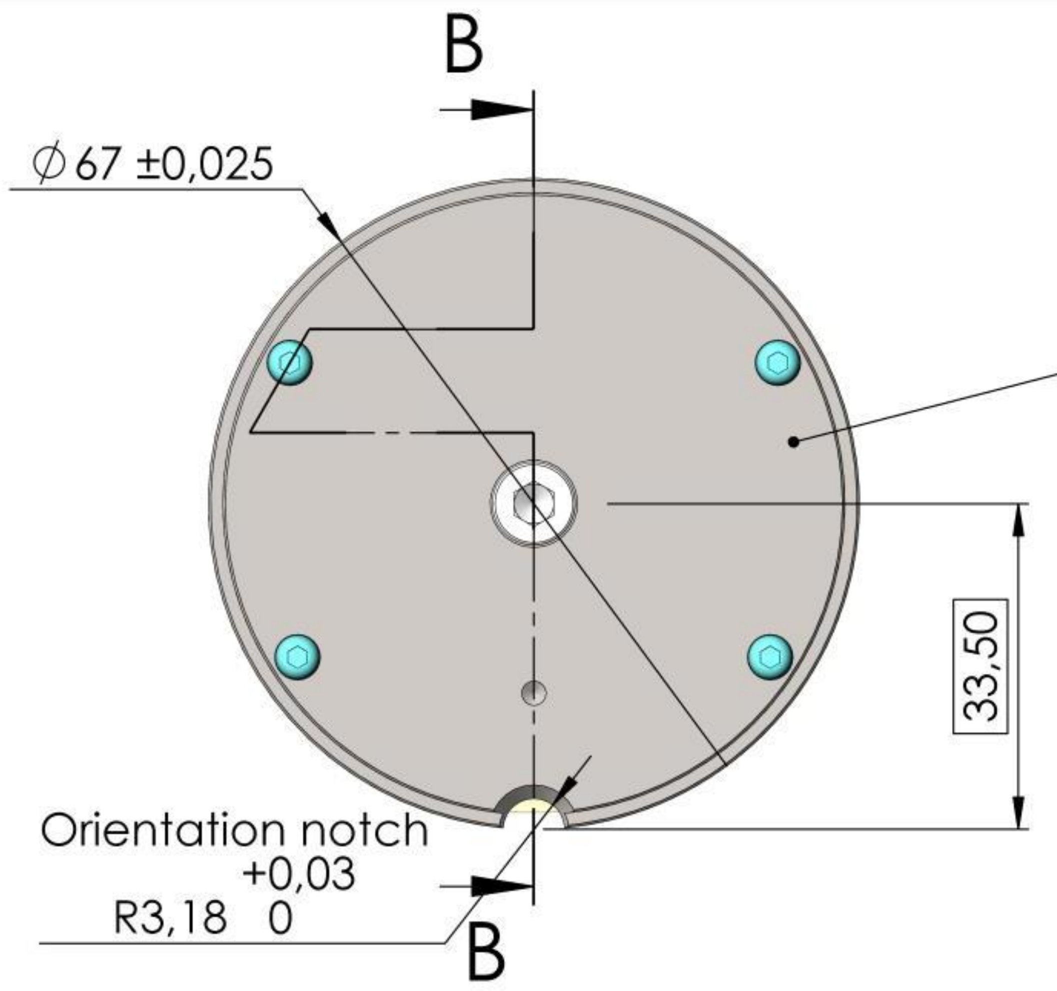

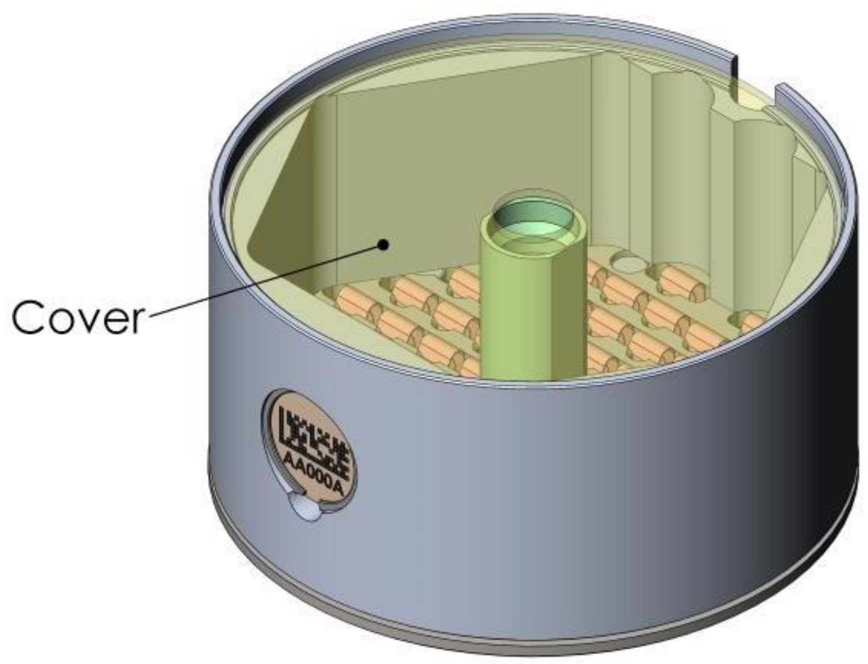

Well	X	Y
1	-6	25,981
2	6	25,981
3	-15	20,785
4	-9	20,785
5	-3	20,785
6	3	20,785
7	9	20,785
8	15	20,785
9	-18	15,588
10	-12	15,588
11	-6	15,588
12	0	15,588
13	6	15,588
14	12	15,588
15	18	15,588
16	-21	10,392
17	-15	10,392
18	-9	10,392
19	-3	10,392
20	3	10,392
21	9	10,392
22	15	10,392
23	21	10,392

Well	X	Y
24	-24	5,196
25	-18	5,196
26	-12	5,196
27	12	5,196
28	18	5,196
29	24	5,196
30	-27	0
31	-21	0
32	-15	0
33	-9	0
34	9	0
35	15	0
36	21	0
37	27	0
38	-24	-5,196
39	-18	-5,196
40	-12	-5,196
41	12	-5,196
42	18	-5,196
43	24	-5,196
44	-21	-10,392
45	-15	-10,392
46	-9	-10,392

Well	X	Y
47	-3	-10,392
48	3	-10,392
49	9	-10,392
50	15	-10,392
51	21	-10,392
52	-18	-15,588
53	-12	-15,588
54	-6	-15,588
55	0	-15,588
56	6	-15,588
57	12	-15,588
58	18	-15,588
59	-15	-20,785
60	-9	-20,785
61	-3	-20,785
62	3	-20,785
63	9	-20,785
64	15	-20,785

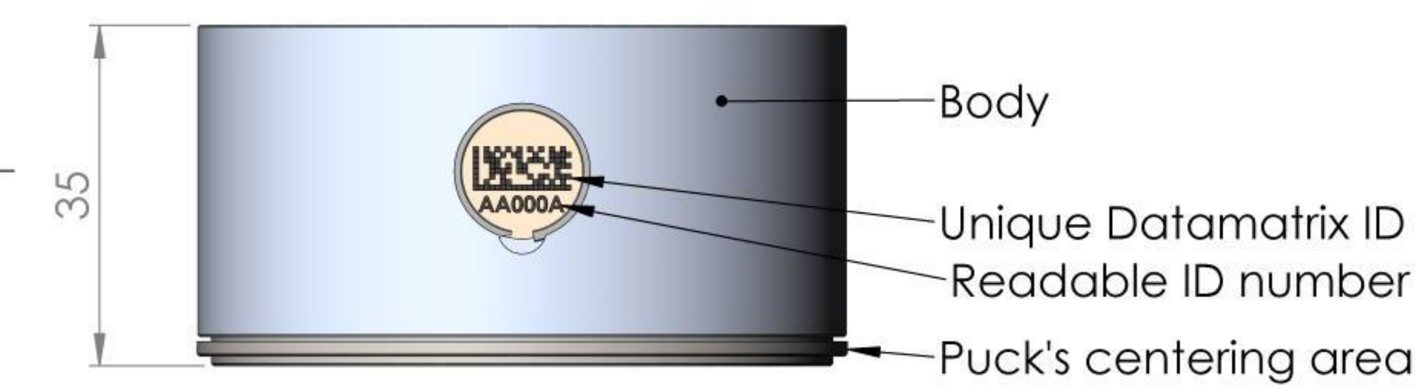

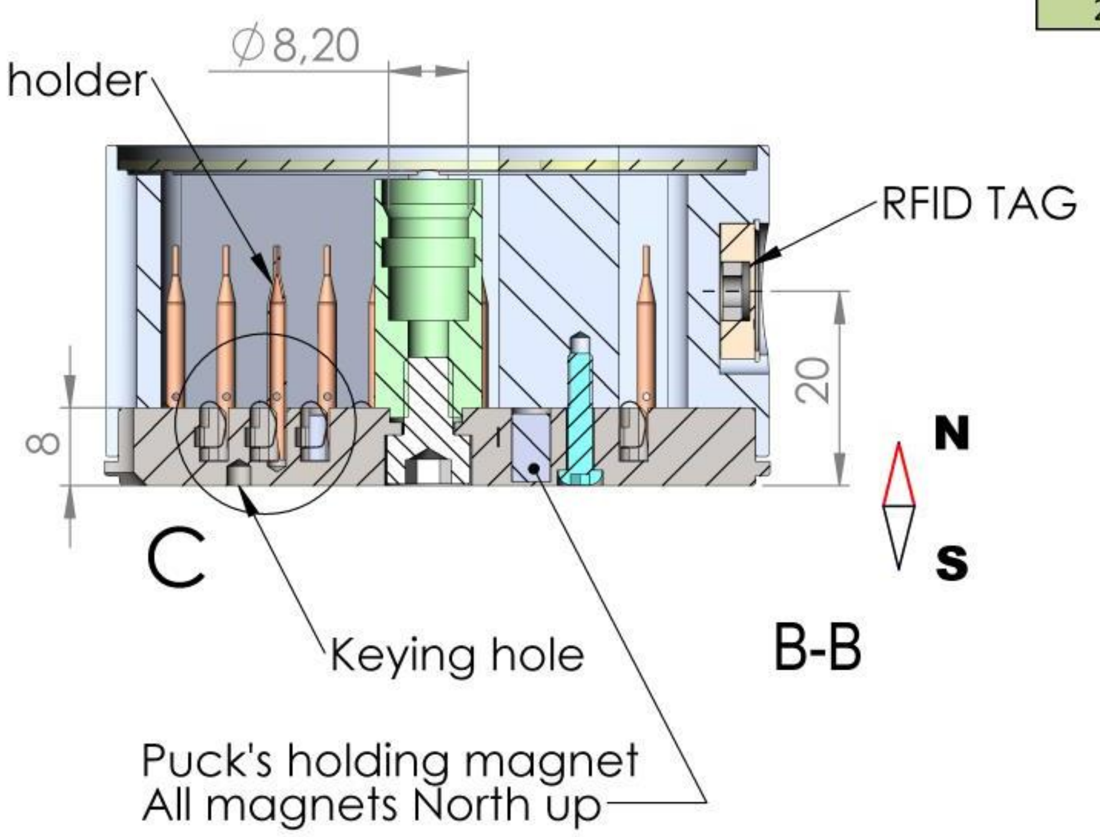

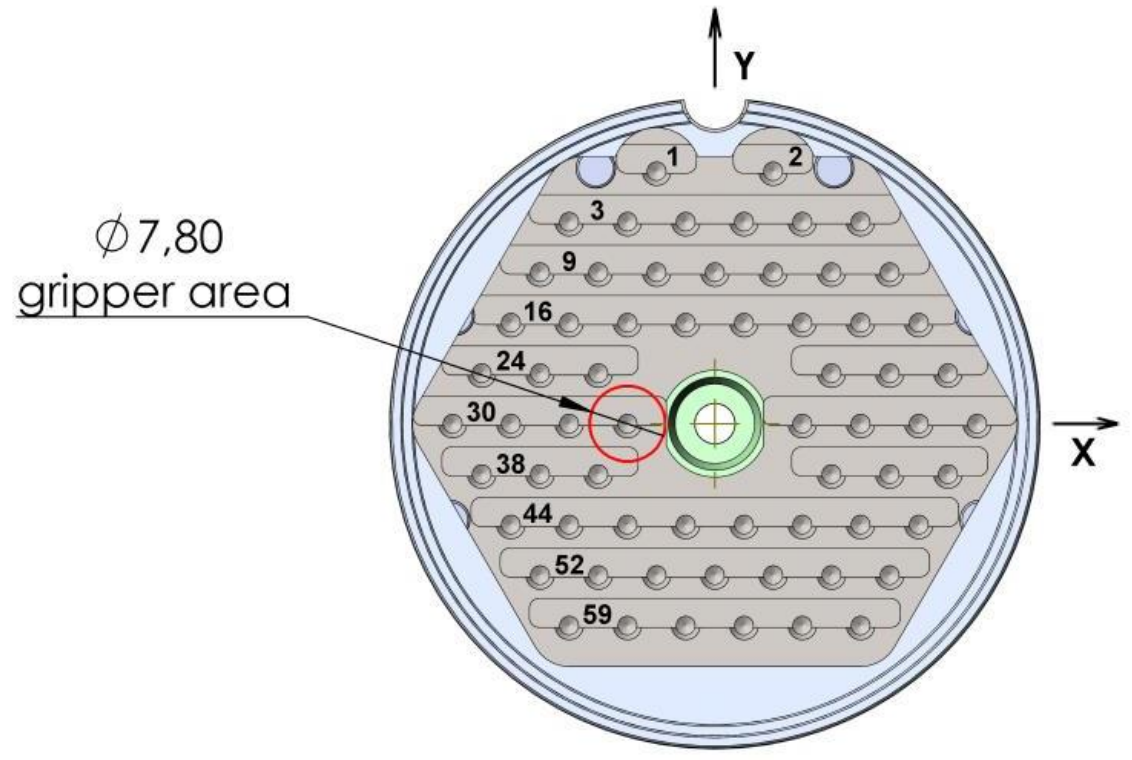

Wells identification and coordinates (coordinates of the pins)

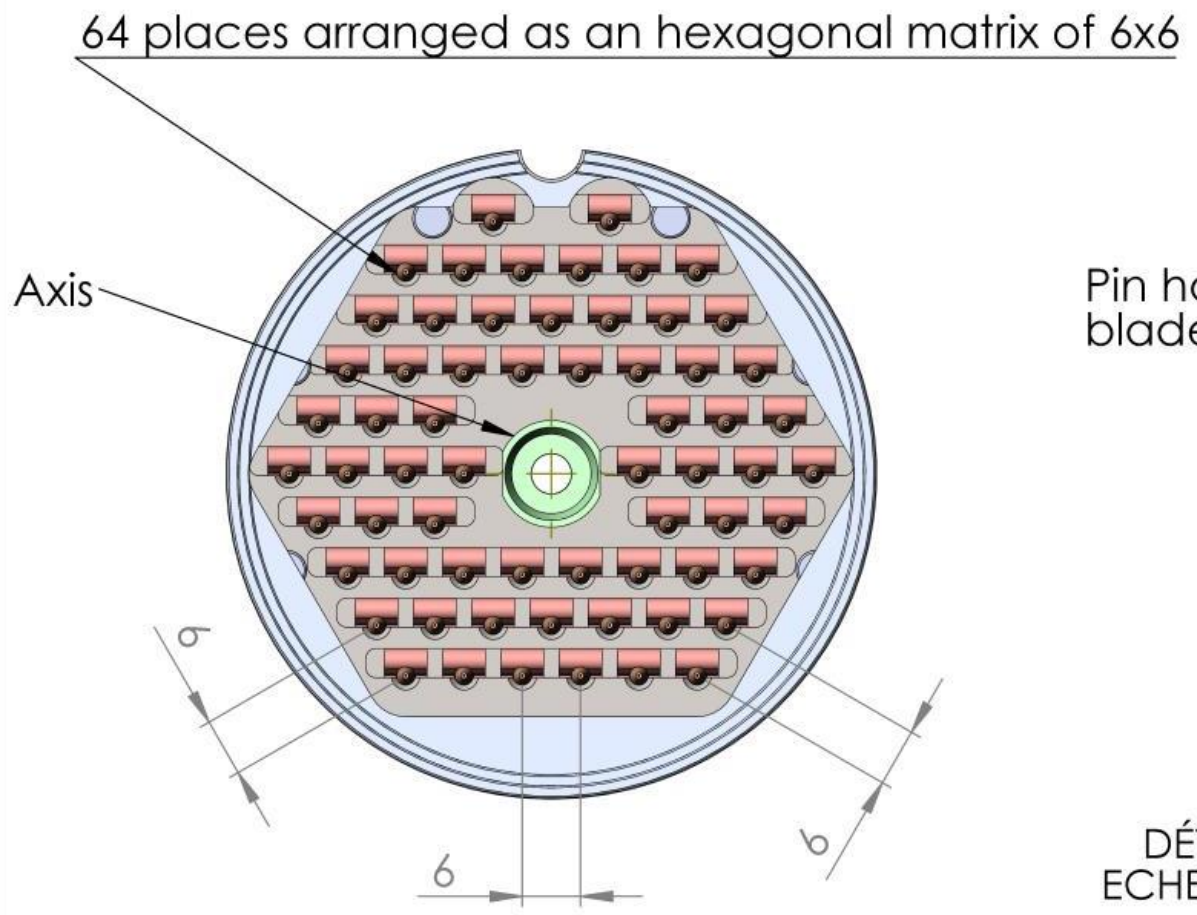

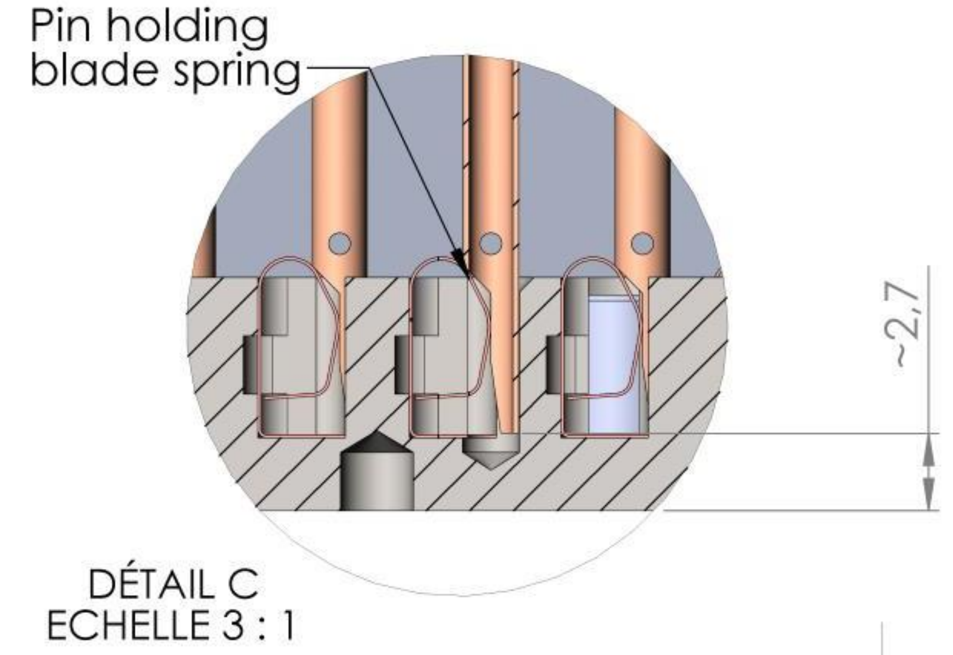

DÉTAIL C
ECHELLE 3 : 1

Preliminary information

	Date	Modification	Auteur
c			
b			
a			

Titre

NewPin Puck 64 Beta1

Ce dessin est la propriété exclusive de EMBL toute reproduction ou utilisation de l'objet représenté sont interdits sans autorisation

Numéro - Indice de mise à jour
11121 100

Projet : NewPin

Sous-ensemble : NewPin puck 64

Matière :

Remarque :

Qté : 1

Dessiné : C.ROSSI

Date : 14/03/2017

Echelle : 1:1

EMBL
Antenne de Grenoble
Laboratoire Européen de Biologie Moléculaire
71av des Martyrs - 38000 Grenoble
téléphone 0 476 207 188 - fax 0 476 207 199

Format A3
