# Supplementary material for: Towards a compact and precise sample holder for macromolecular crystallography
Source: Acta Crystallogr D Struct Biol. 2017 Sep 29;73(Pt 10):829–40. doi: 10.1107/S2059798317013742 (PMC5633908; doi:10.1107/S2059798317013742)
Supplement: Supplementary file 3 [file d-73-00829-sup3.pdf]

STRUCTURAL
BIOLOGY

Volume 73 (2017)

Supporting information for article:

Towards a compact and precise sample holder for macromolecular crystallography

Gergely Papp, Christopher Rossi, Robert Janocha, Clement Sorez, Marcos Lopez-Marrero, Anthony Astruc, Andrew McCarthy, Hassan Belrhali, Matthew W. Bowler and Florent Cipriani

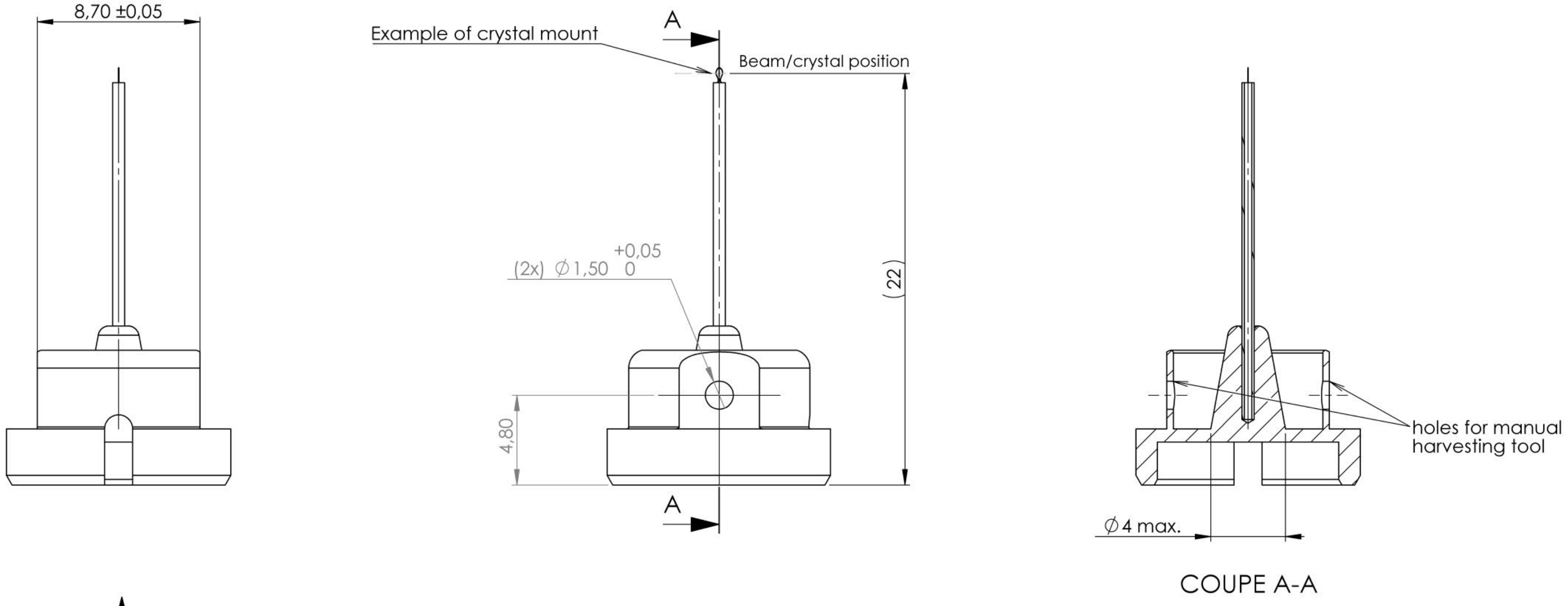

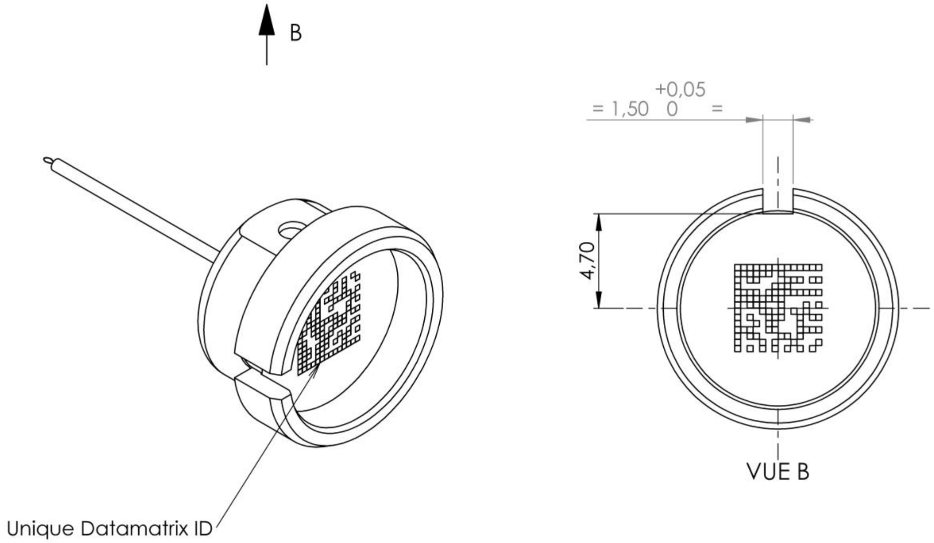

Preliminary information

Refer to SPINE standard for other dimension

c			
b			
a			
	Date	Modification	Auteur

Titre<br>**SPINEplus Sample holder V1**<br>**(base+needle)**		Ce dessin est la propriété exclusive de EMBL toute reproduction ou utilisation de l'objet représenté sont interdits sans autorisation<br>Tolérances générales (sauf indications)<br>**JS10 - Ra 1,6 - angles cassés à 0,2mm**
Numéro - Indice de mise à jour<br>**1141 100**	Projet : NewPin<br>Sous-ensemble : SPINEplus Sample holder	**EMBL**<br>**Antenne de Grenoble**
Matière :	Remarque : / Qté :	**Laboratoire Européen de Biologie Moléculaire**
Dessiné : C.ROSSI	Date : 21/03/2017 / Echelle : 4:1	71av des Martyrs - 38000 Grenoble<br>téléphone 0 476 207 188 - fax 0 476 207 199

Format A3

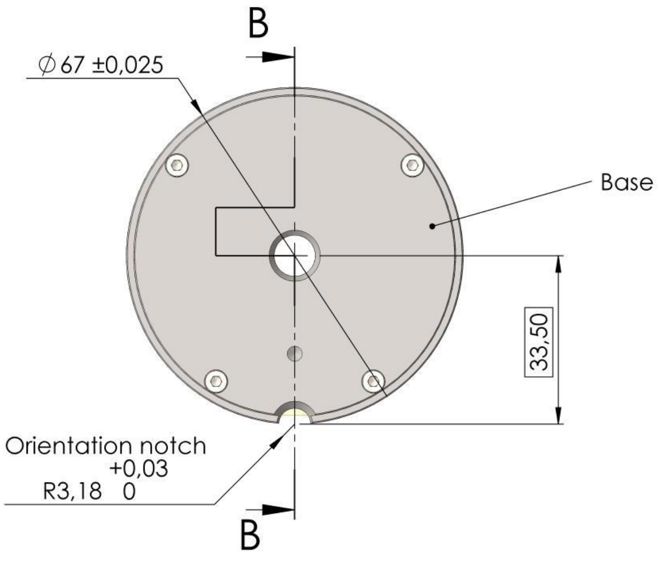

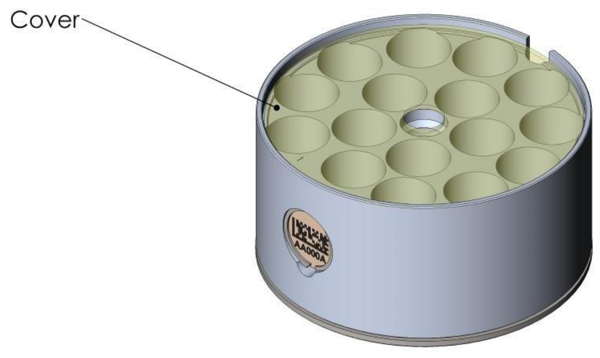

Well	X	Y
1	-14,93	-20,67
2	-23,8	-9,16
3	-25,14	4,27
4	-18,73	17,31
5	-7,27	24,44
6	7,27	24,44
7	18,73	17,31
8	25,14	4,27
9	23,8	-9,16
10	14,93	-20,67
11	1,44	-21,24
12	-7,23	-9,58
13	-11,45	3,24
14	0,22	11,9
15	11,65	4,71
16	7,7	-9,27

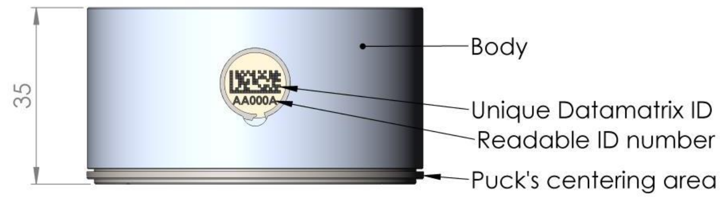

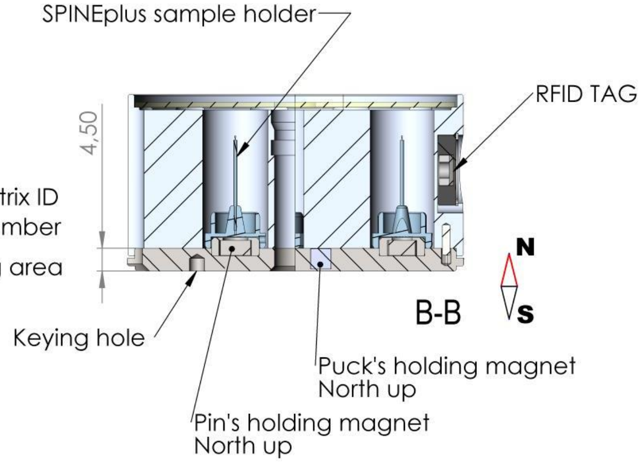

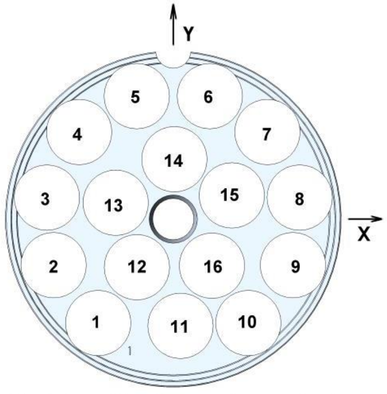

Wells identification and coordinates (coordinates of the pins)

16 wells arranged for double gripper compatibility

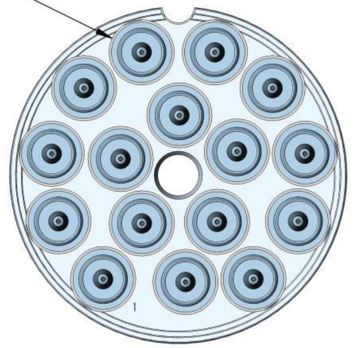

Preliminary information

	Date	Modification	Auteur
c			
b			
a	14/03/2017		C.ROSSI

Titre

SPINEplus puck Beta1

Ce dessin est la propriété exclusive de EMBL toute reproduction ou utilisation de l'objet représenté sont interdits sans autorisation

Numéro - Indice de mise à jour
1131 00-A

Projet : NewPin
Sous-ensemble : SPINEplus puck
Matière : -
Remarque :
Qté :
Dessiné : C.ROSSI
Date : 10/01/2016
Echelle : 1:1

EMBL
Antenne de Grenoble
Laboratoire Européen de Biologie Moléculaire
71av des Martyrs - 38000 Grenoble
téléphone 0 476 207 188 - fax 0 476 207 199

Format A3
