# Supplementary material for: Towards a compact and precise sample holder for macromolecular crystallography
Source: Acta Crystallogr D Struct Biol. 2017 Sep 29;73(Pt 10):829–40. doi: 10.1107/S2059798317013742 (PMC5633908; doi:10.1107/S2059798317013742)
Supplement: Supplementary file 4 [file d-73-00829-sup4.pdf]

STRUCTURAL
BIOLOGY

Volume 73 (2017)

Supporting information for article:

Towards a compact and precise sample holder for macromolecular crystallography

Gergely Papp, Christopher Rossi, Robert Janocha, Clement Sorez, Marcos Lopez-Marrero, Anthony Astruc, Andrew McCarthy, Hassan Belrhali, Matthew W. Bowler and Florent Cipriani

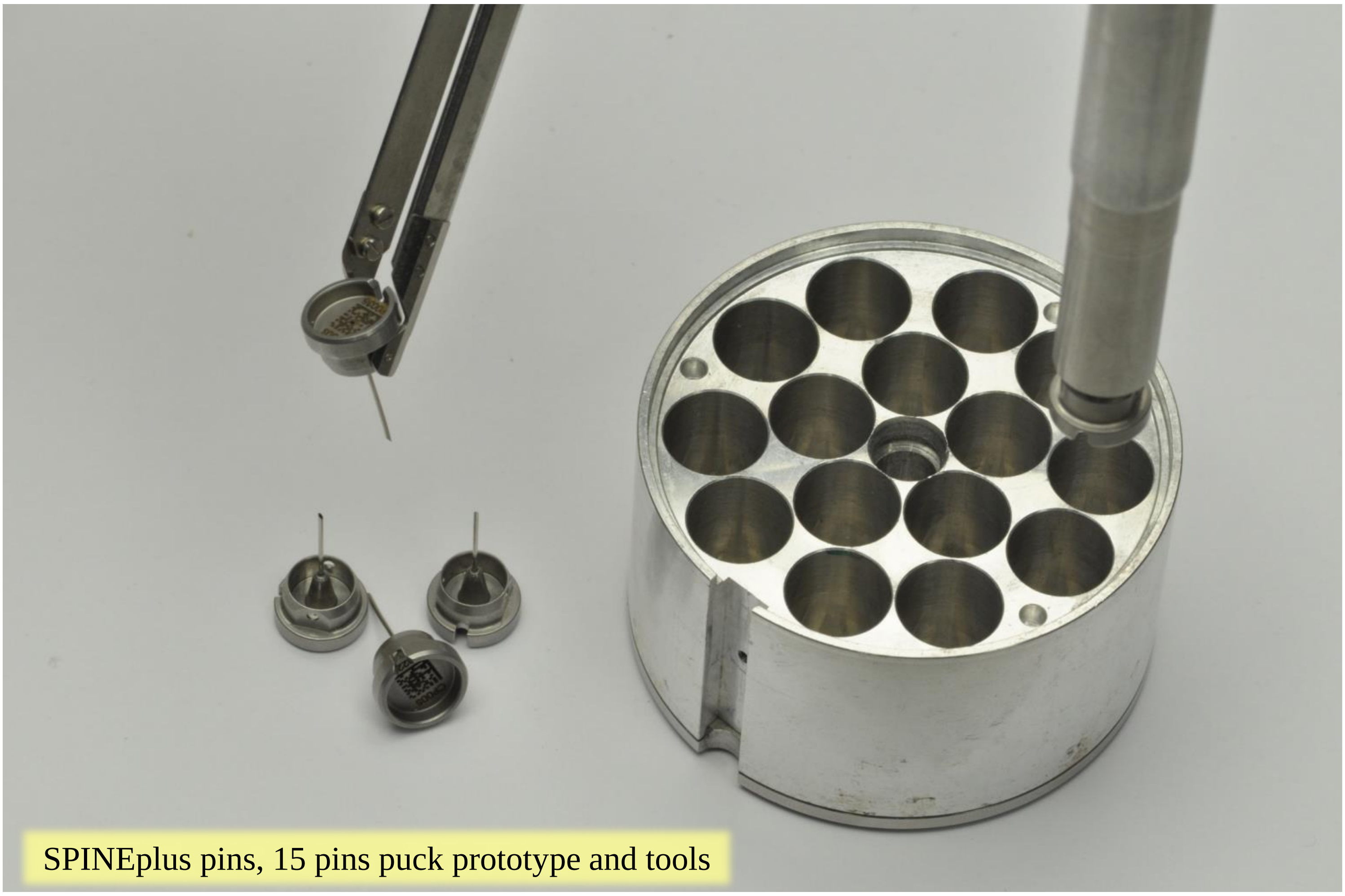

Figure S 1: SPINEplus sample holder, puck 15 slots and manual handling tools (picture)

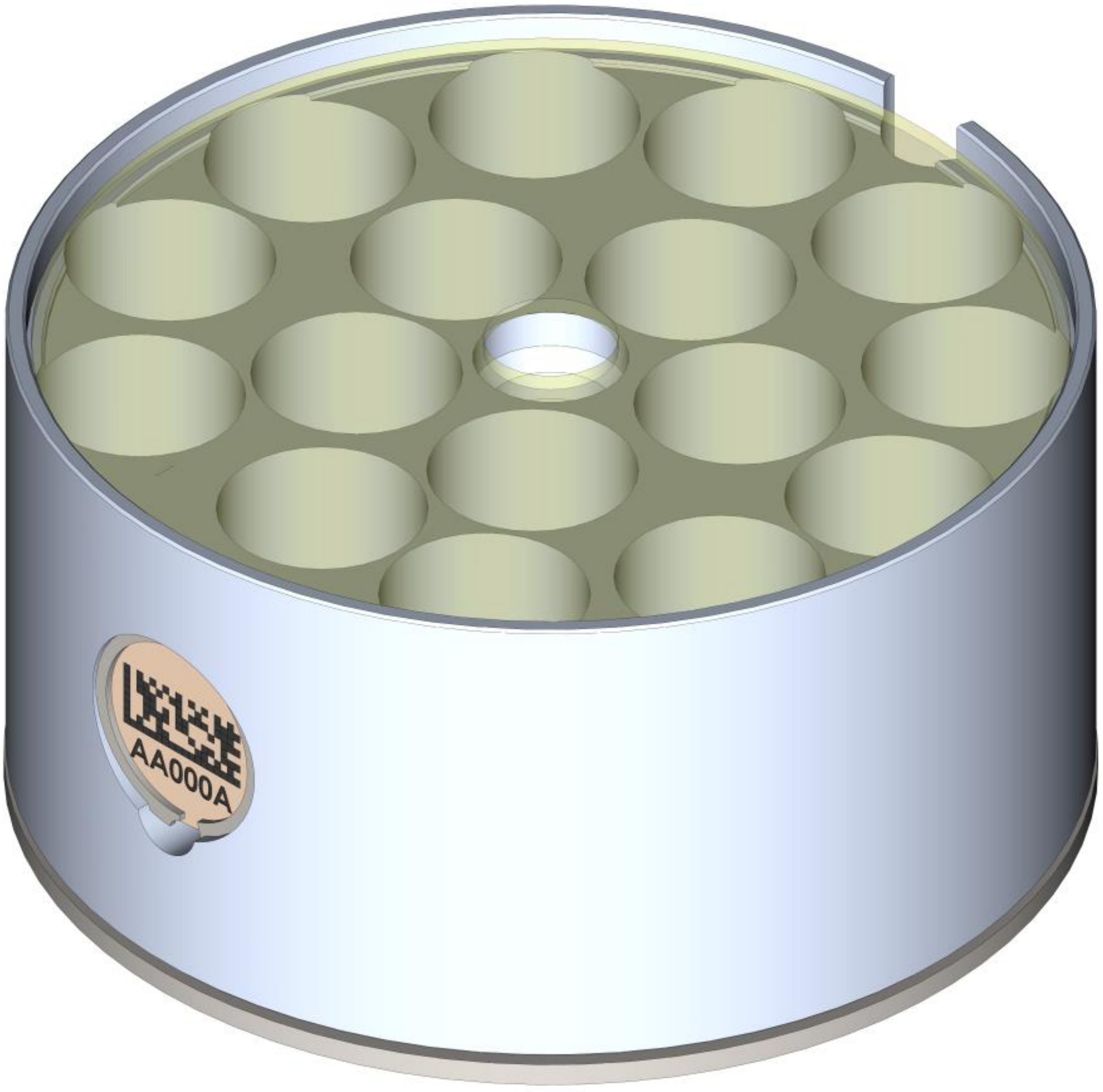

SPINEplus puck Beta1

Figure S 2: SPINEplus puck 16 slots (3D view)

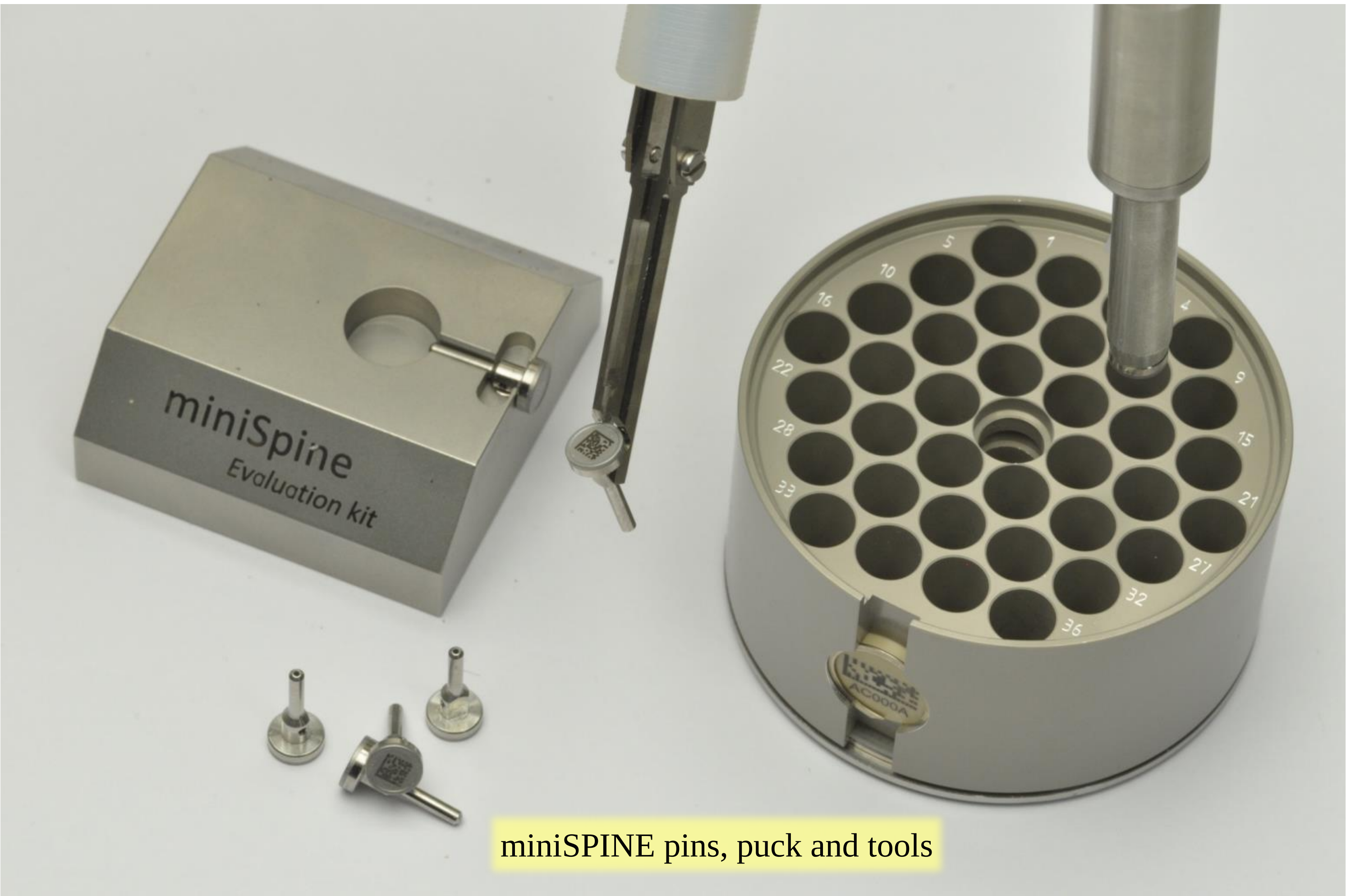

Figure S 3: miniSPINE sample holders, puck and manual handling tools (picture)

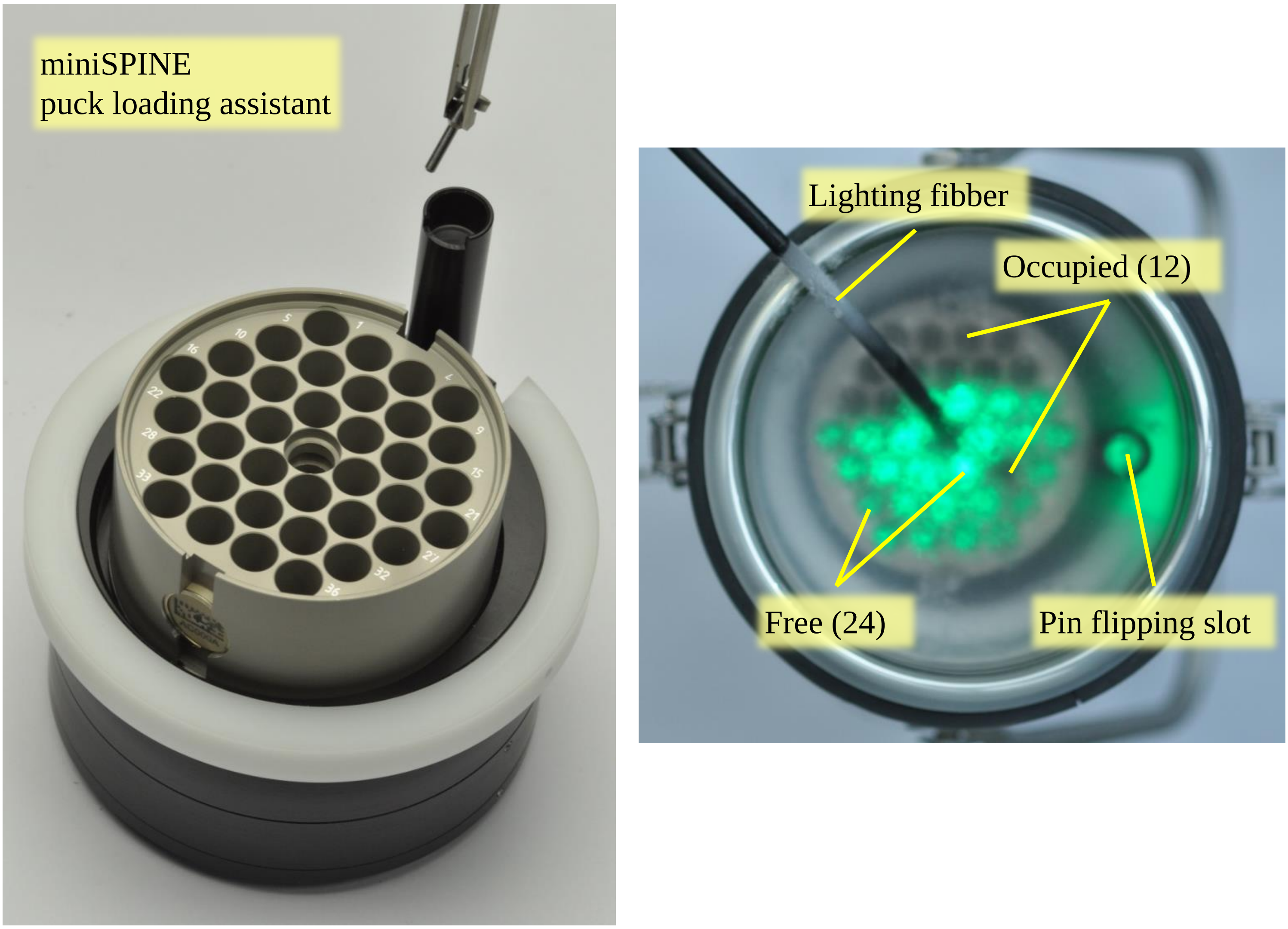

Figure S 4: miniSPINE puck filling assistant (picture)

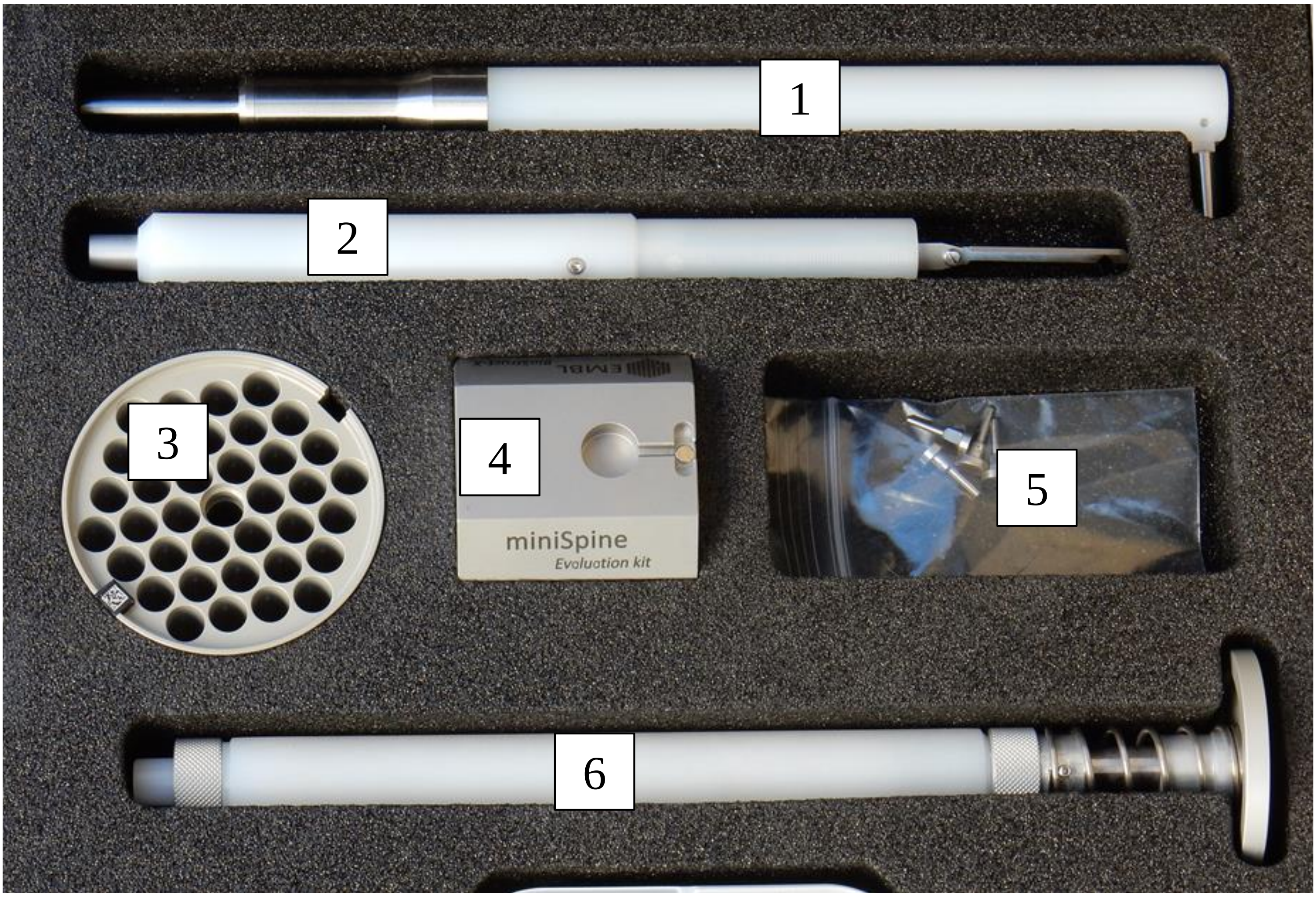

Figure S 5: miniSPINE manual handling evaluation kit (picture): Pin extraction tool (1), manual harvesting tool (2), puck (3), harvesting assistant (4), sample holders (5) and puck handling tool (6)

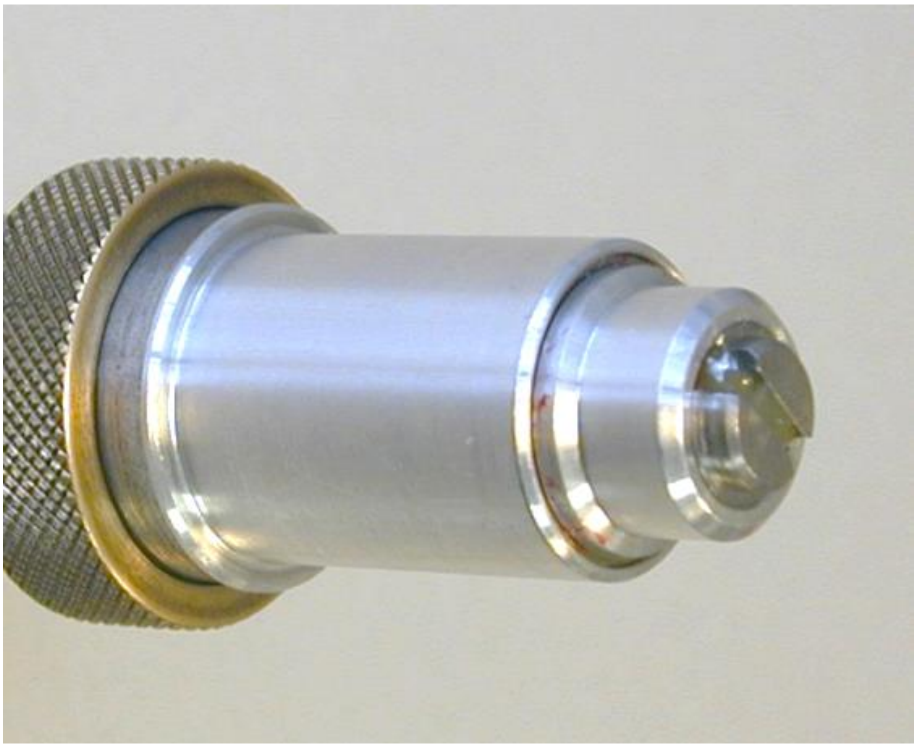

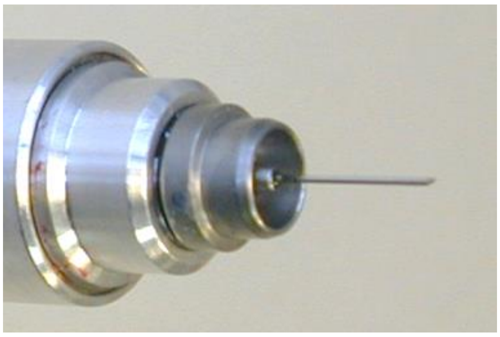

SPINE pin

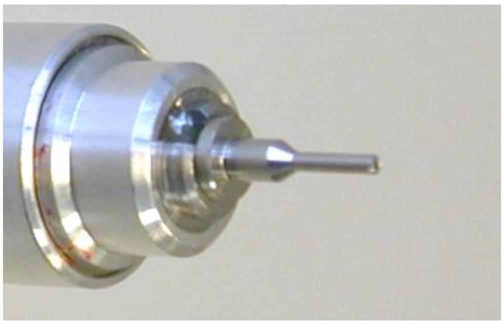

SPINE & miniSPINE SmartMagnet head

miniSPINE pin

Figure S 6: miniSPINE/SPINE SmartMagnetP (picture)

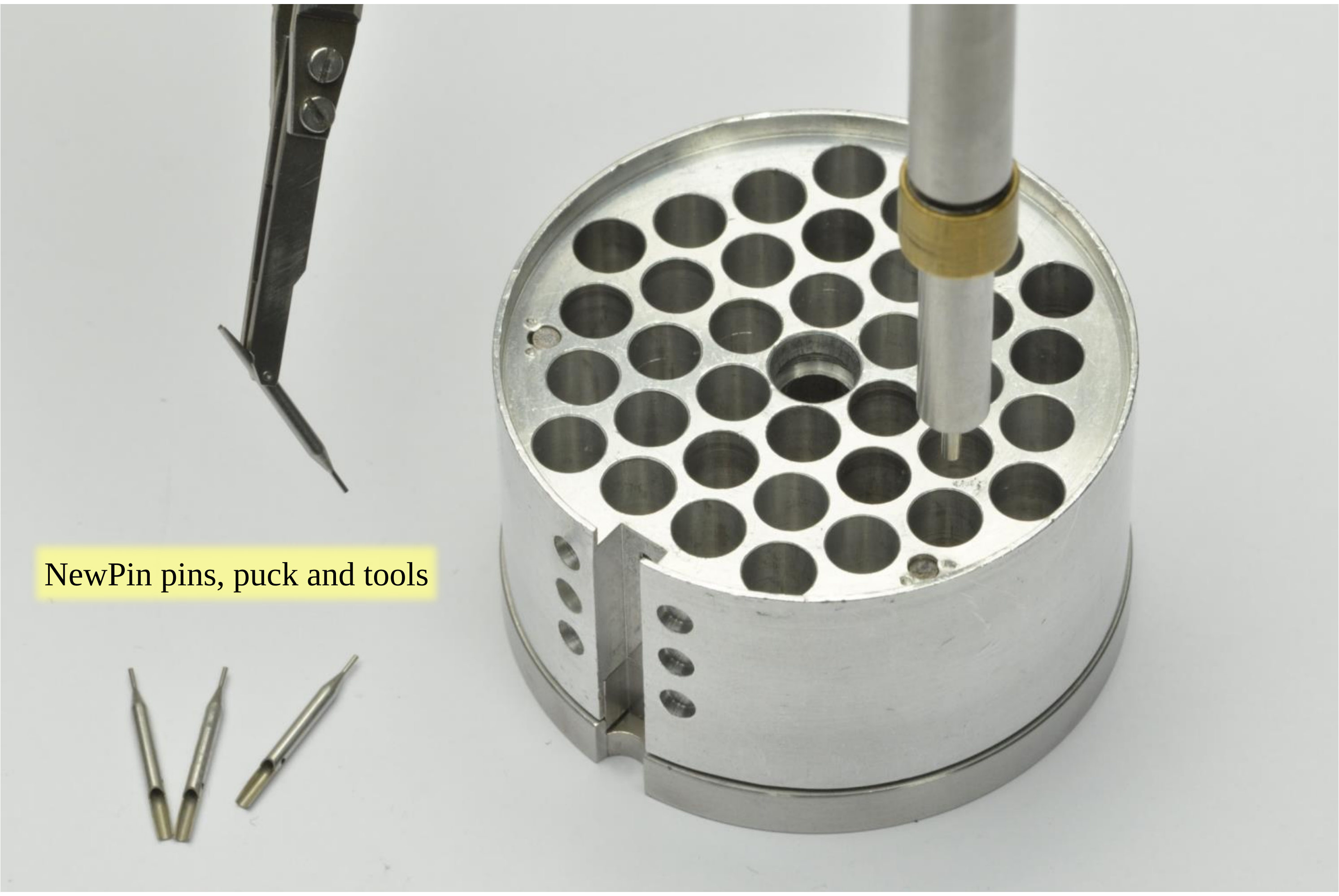

Figure S 7: NewPin sample holder, puck and manual handling tools (picture)

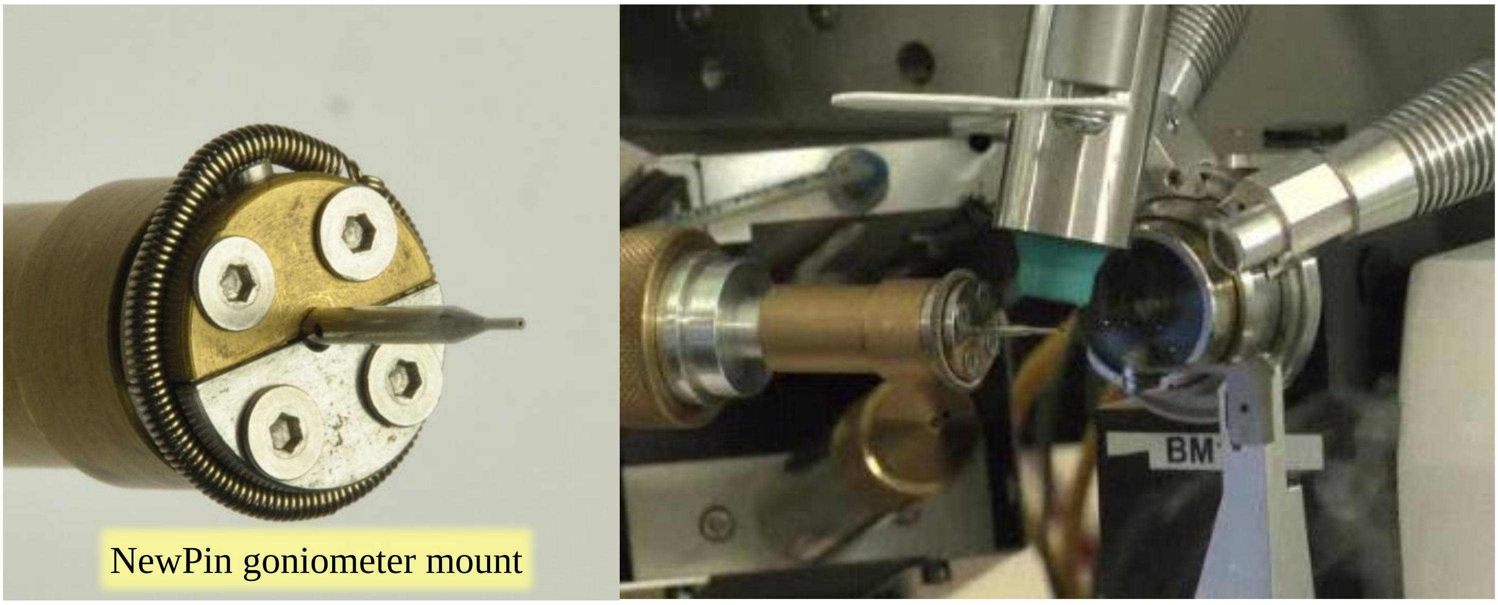

Figure S 8: NewPin goniometer mount (picture)

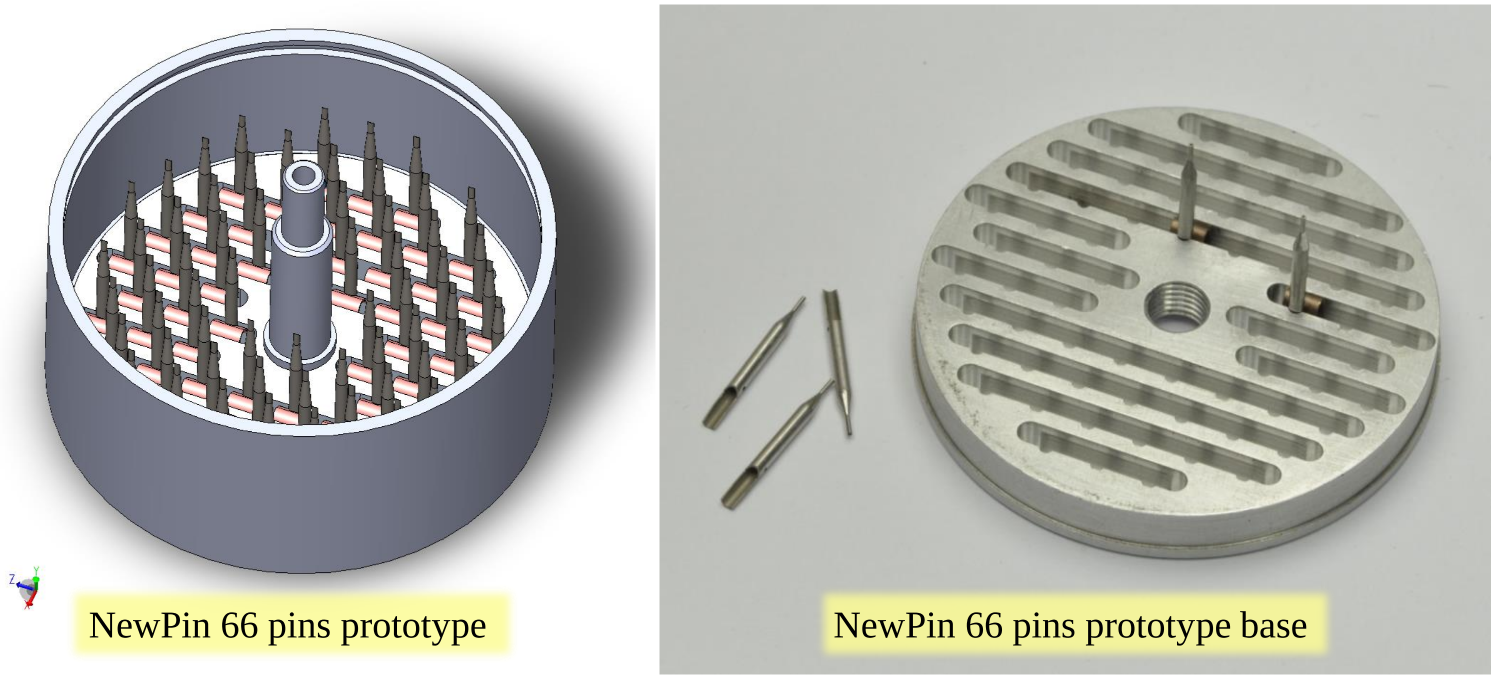

Figure S 9: NewPin puck 66 pins prototype (picture)

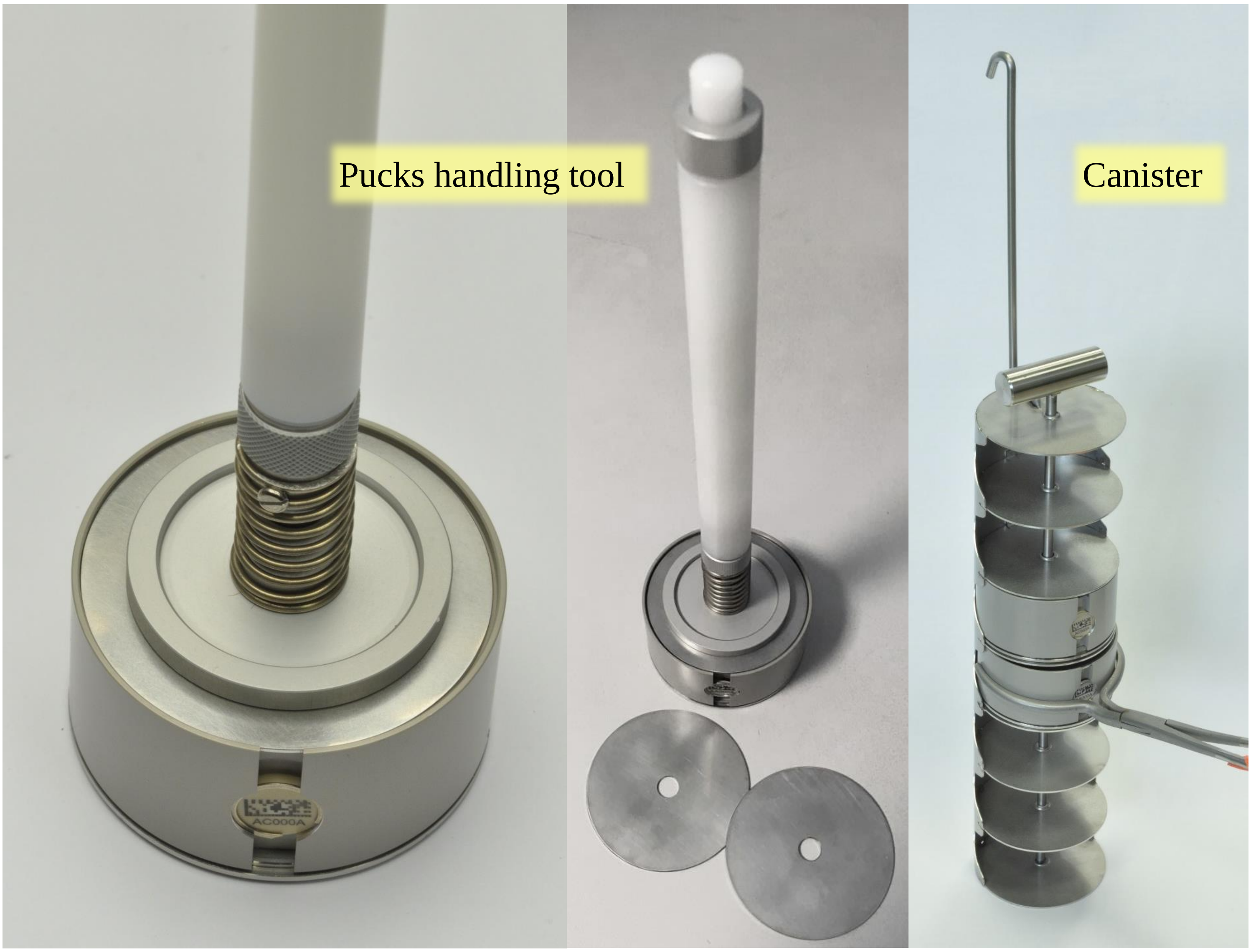

Figure S 10: Pucks handling tool and 7 puck shelved canister (picture)

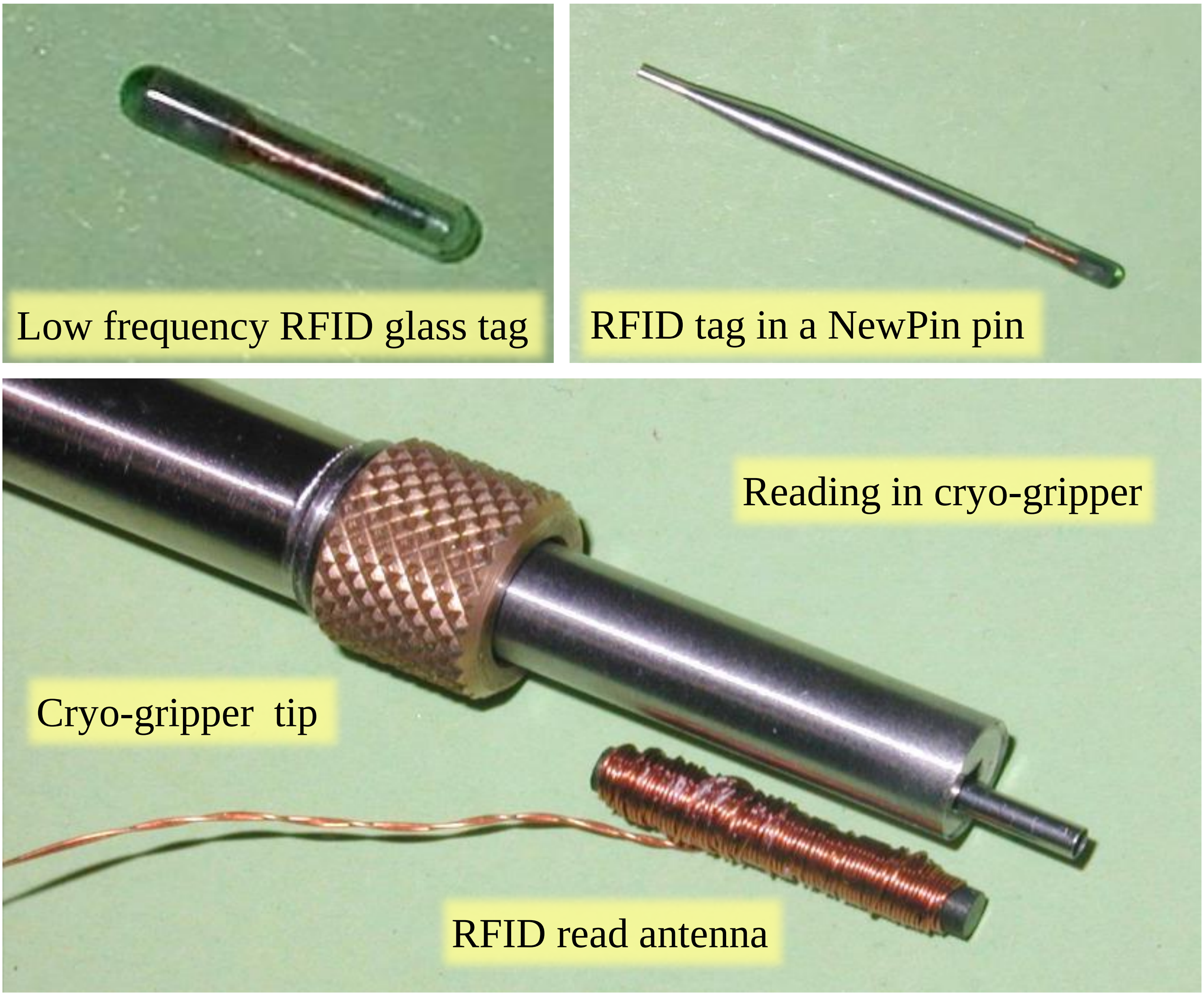

Figure S 11: miniSPINE MSrf and NewPin RFID identification: RFID glass tag , NewPin prototype with RFID glass tag inside , NewPin/miniSpine robot gripper prototype with tagged NewPin Pin inside and RFID reader antenna

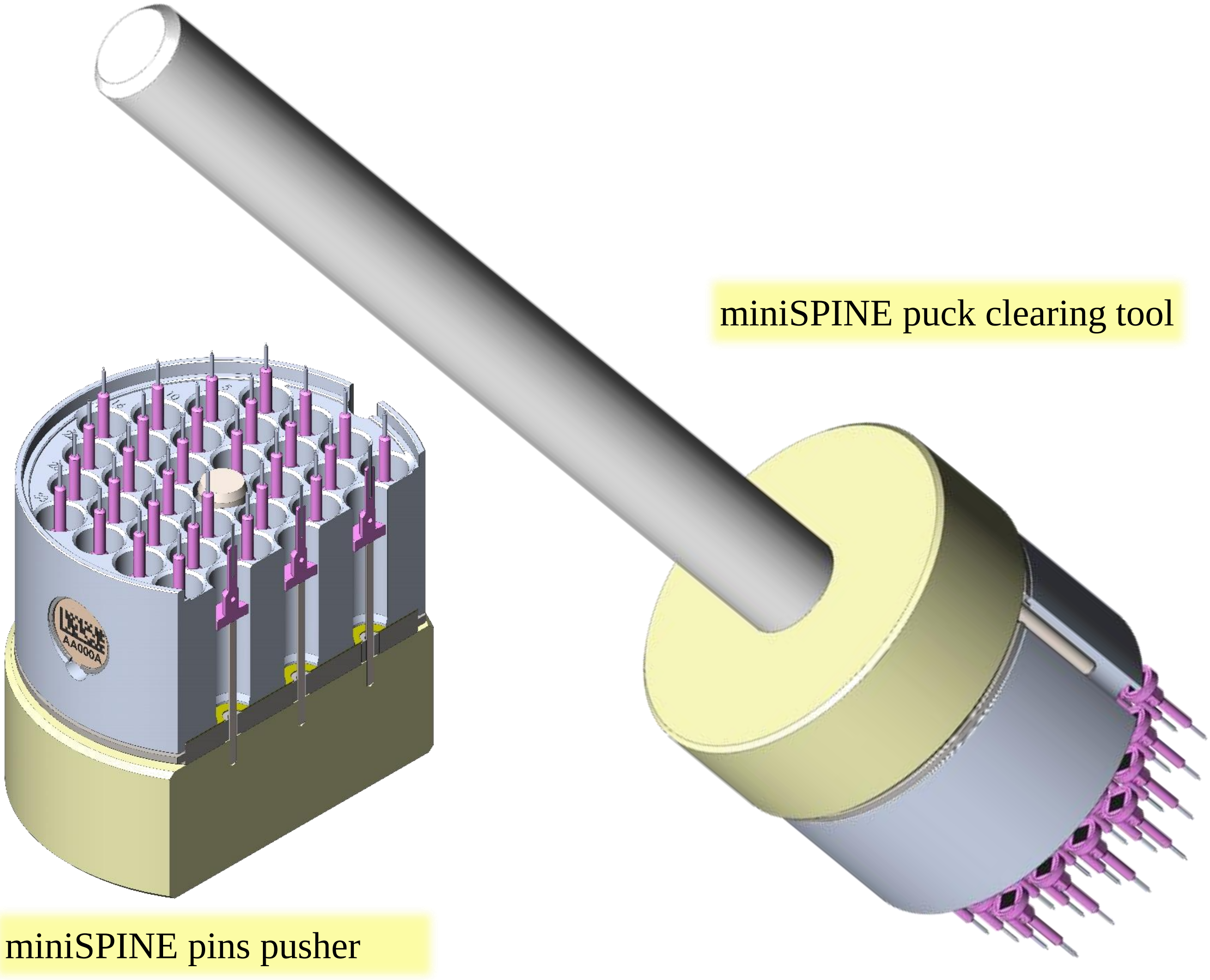

Figure S 12: miniSPINE pins pusher and puck clearing tool

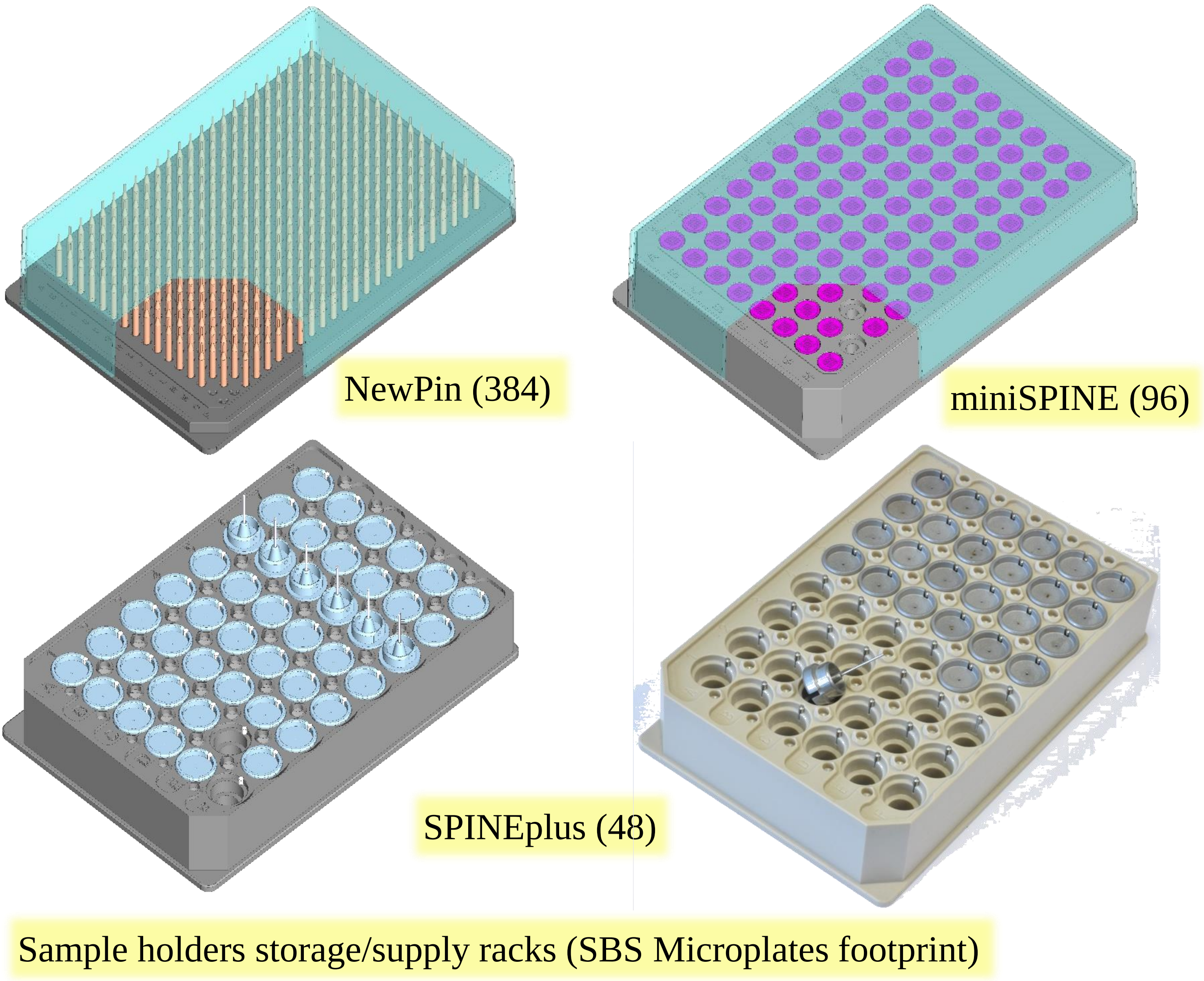

Figure S 13: Examples of storage racks with pin orientation for NewPin, miniSPINE and SPINEplus sample holders
